# Incorporating platinum circular economy into China’s hydrogen pathways toward carbon neutrality

**DOI:** 10.1093/pnasnexus/pgae172

**Published:** 2024-05-14

**Authors:** Peng Wang, Chenyang Wang, Jiashuo Li, Klaus Hubacek, Laixiang Sun, Fan Yang, Kuishuang Feng, Wei-Qiang Chen

**Affiliations:** Key Lab of Urban Environment and Health, Institute of Urban Environment, Chinese Academy of Sciences, Xiamen 361021, China; College of Resources and Environment, University of Chinese Academy of Sciences, Beijing, China; Key Lab of Urban Environment and Health, Institute of Urban Environment, Chinese Academy of Sciences, Xiamen 361021, China; Robert M. Buchan Department of Mining, Smith Engineering, Queen's University, Kingston, ON, K7L 3N6, Canada; Institute of Blue and Green Development, Shandong University, Weihai 264209, China; Integrated Research on Energy, Environment and Society (IREES), Energy and Sustainability Research Institute Groningen, University of Groningen, Groningen 9747 AG, the Netherlands; Department of Geographical Sciences, University of Maryland, College Park, MD 20742, USA; Institute of Blue and Green Development, Shandong University, Weihai 264209, China; Department of Planning, Aalborg University, Aalborg 9000, Denmark; Department of Geographical Sciences, University of Maryland, College Park, MD 20742, USA; Key Lab of Urban Environment and Health, Institute of Urban Environment, Chinese Academy of Sciences, Xiamen 361021, China; College of Resources and Environment, University of Chinese Academy of Sciences, Beijing, China

**Keywords:** carbon neutrality, material flow analysis, hydrogen, platinum, circular economy

## Abstract

Hydrogen is gaining tremendous traction in China as the fuel of the future to support the country’s carbon neutrality ambition. Despite that hydrogen as fuel largely hinges on the supply of platinum (Pt), the dynamic interlinkage between Pt supply challenges, hydrogen development pathways, and climate targets in China has yet to be deeply analyzed. Here, we adopt an integrated assessment model to address this important concern and corresponding strategies for China. The results indicate that the booming hydrogen development would drive China’s cumulative demand for Pt metal to reach 4,200–5,000 tons. Much of this demand, met through a limited supply pattern, is vulnerable to price volatility and heightened geopolitical risks, which can be mitigated through circular economy strategies. Consequently, a coordinated approach to leverage both global sustainable Pt sourcing and a robust domestic Pt circular economy is imperative for ensuring cost-effective hydrogen production, aligned with a climate-safe future.

**Significance Statement**

We have employed an integrated assessment model to explore this nexus and found that China’s hydrogen demand could impose substantial requirements for Pt metal, with a heavy dependence on imports, placing it within the context of price volatility and significant geopolitical risks. Our analysis of circular economy strategies underscores their potential in mitigating these associated uncertainties. Simultaneously, we emphasize the urgent need for collaborative efforts on a global scale to ensure sustainable Pt sourcing, in addition to enhancing domestic material efficiency. These efforts are essential for China to address Pt supply risks and achieve its hydrogen ambitions toward carbon neutrality.

## Introduction

Hydrogen (H_2_) is a versatile, storable, and energy-dense fuel that can help to tackle various critical energy challenges toward a low-carbon future (1–3). Hydrogen can be generated from various feedstocks (such as hydropower, fossil fuels, biomass, wind, solar, and geothermal), converted back to electricity through fuel cells, and transported over long distances (4). The growing concerns on global warming and geopolitical tensions further make hydrogen-fuel gain political traction as an imperative strategy to achieve national energy security and carbon neutrality (CN) targets (2, 3, 5). China, as the world’s largest CO_2_ emitter, is keen to develop clean, affordable, and reliable energy systems to meet diverse national needs (e.g. long-distance mobility and energy supply to sectors facing tough decarbonization challenges, such as heavy industry) (3, 6), and expects hydrogen to play a significant role in both safeguarding China’s energy security and protecting the environment (7–9). Indeed, China has integrated the development of hydrogen energy industry chains as one core component of its 2060 CN package, with the expectation that hydrogen would comprise more than 10% of total energy supply by 2050 (10–12) (Table S1). China has also commissioned the world's largest green-hydrogen project and announced more than 50 large-scale hydrogen projects, with an investment value of over $180 billion. About 50% of these announced projects will support green transport, a key transition for achieving CN by 2060 (13).

A critical issue arises herewith is platinum (Pt) as one critical potential bottleneck for the large-scale deployment of high-performance green-hydrogen infrastructure. Despite some substitution potentials, Pt is widely considered the most efficient material to trigger the hydrogen evolution reaction to achieve high reaction rates in the process of water electrolysis (14). Meanwhile, the electrode reactions rely heavily on the use of Pt catalysts for the reduction of oxygen in the most common types of hydrogen-fuel cells (15). Indeed, the more efficient and cleaner the hydrogen value chain is, the more Pt is needed (16, 17). However, Pt is one of the most precious and scarcest metals in the world, with a production quantity equal to only around 6% of that of gold in 2020 (18); and the geographical distribution of Pt is highly concentrated, with 91% of known reserves in South Africa (19–21). As the world’s largest Pt consumer, China is highly vulnerable to the international Pt supply interruptions because over 95% of primary Pt input is sourced from foreign countries (19). This high dependency of foreign supply has stimulated a growing concern over platinum supply security amid the development and expansion of hydrogen-fuel value chains (especially for fuel cells) within China and beyond (15, 22–27).

Recently, a series of reports produced by various international organizations ranging from IEA (28), World Bank (29), IRENE (30), to WWF (31) have highlighted the potential tensions between the expected increase in demand for hydrogen fuel and the constraints of the availability of critical raw materials on the paths of national and global low-carbon transitions. Assessment approaches employed in these reports and other publications include dynamic material flow analysis (MFA) (23, 24), input–output analysis (32), system dynamics (33), and time-series analysis (27, 34–36). The estimated future platinum demands are typically presented at the global level, and some related results are summarized in Table S2. There is a lack of attention to a nation-specific concern on the Pt supply constraints, with the only exception of a case study on Germany by Kiemel et al. (37). More importantly, different climate targets can also significantly alter hydrogen infrastructure trajectories (38–40), as the corresponding platinum requirements are affected by different pathways of alternative technologies. This makes the joint assessment of Pt supply chains, hydrogen energy industrial chains, and the climate targets crucial for a systematic understanding of the challenges and for informing joint measures to mitigate the risk.

In this research, China is exemplified as a representative case for understanding the interplay between its hydrogen ambition and the pressures of platinum (Pt) supply. We map out the potential pathways of China’s hydrogen-fuel industry, charting its course toward CN, from fuel generation to final use. Notably, our focus is solely on hydrogen's role for energy, excluding other applications such as chemical feedstocks which have a negligible demand for Pt (41). We perform such an analysis using an integrated assessment model, Global Change Assessment Model (GCAM), to map the development of China’s hydrogen-fuel industry in the future under three scenarios, namely, business as usual (BAU), 1.5°C warming target (1.5°C), and CN scenario by 2060. Through the investigation of Pt usage in various hydrogen technologies, we further apply dynamic MFA to simulate the changes in supply chain of platinum over its life cycle from mining, trade, throughout to final recycling within China, with specific consideration of recycling potential, material substitution, rival demand from other sectors, and other potential bottlenecks. The results indicate that the development of China’s hydrogen-fuel industry could be severely constrained by the shortage in Pt supply. This constraint could bring heightened uncertainties concerning the hydrogen needs for China's CN pledge. Employing a variety of circular economy strategies could help mitigate these uncertainties. Nevertheless, China would still need to navigate various geopolitical, environmental, and socioeconomic challenges to secure overseas platinum supply and fulfill its hydrogen-fuel ambition.

## Results

### China’s hydrogen pathways under different climate targets

**Fig. 1.**
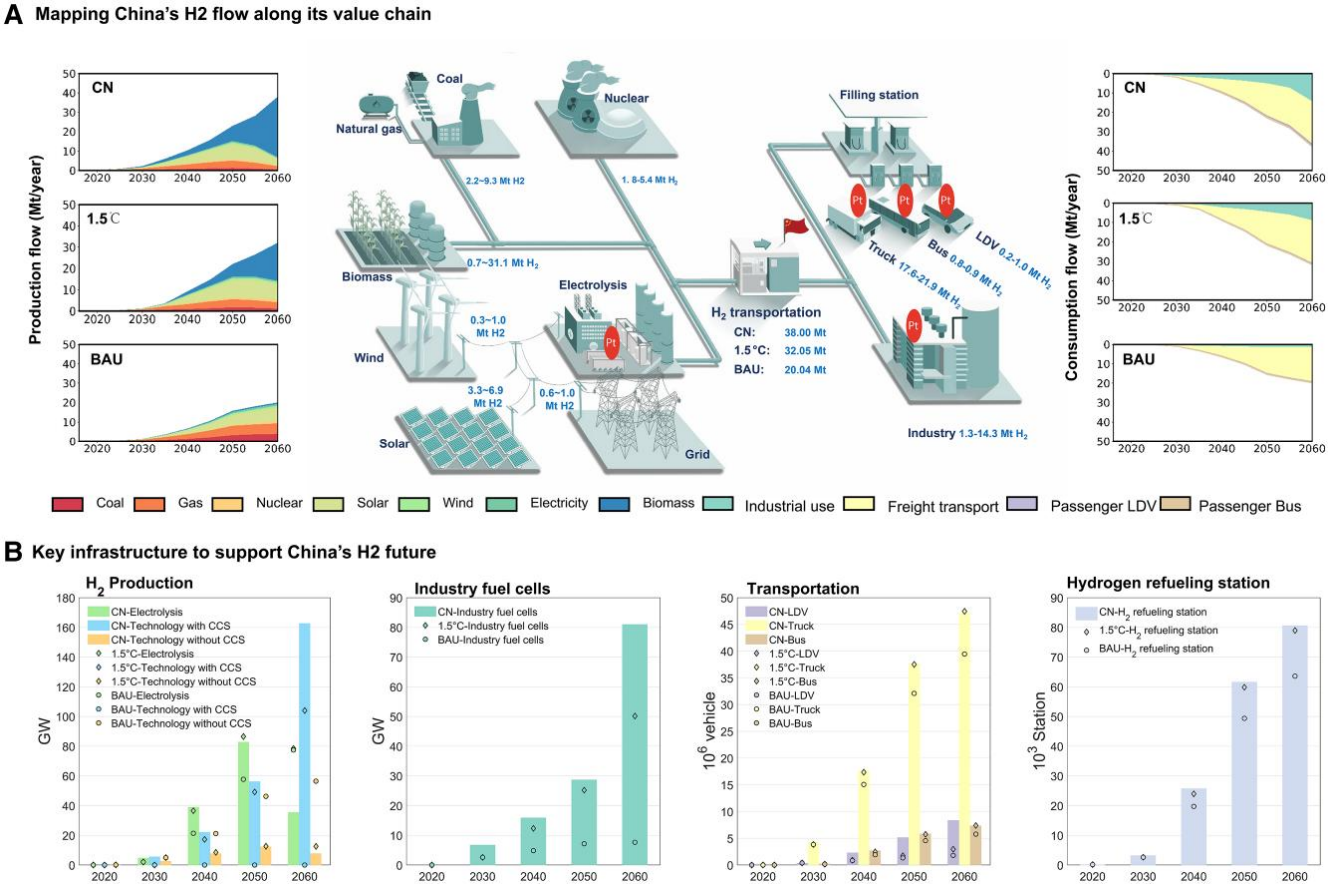
China’s hydrogen-fuel production and consumption mix from 2020 to 2060 under CN, 1.5°C, and BAU scenario. A) China’s hydrogen-fuel future in 2060, with the left-hand side panel showing the evolution of hydrogen production mix from 2020 to 2060 and the right-hand side panel showing the hydrogen consumption structure. Four charts of B map the key infrastructure along the hydrogen supply chain over 2020–2060, including hydrogen production equipment, fuel cells for industry use, fuel cell vehicles, and hydrogen filling stations. Note: This study focuses exclusively on hydrogen as an energy source, not as a chemical feedstock, since the latter does not involve platinum use.

We devise three emission scenarios for China by 2060: (i) the BAU trajectory, where no additional emission regulation policy is enforced during the period from 2020 to 2060; (ii) the 1.5°C trajectory, where China will reduce its emissions through increasing the carbon price by 5% annually from 2040 onward, with a 2040 base price of 208$/ton carbon (Table S3), in order to limit global temperature rise below 1.5°C; and (iii) the CN trajectory, where China will reach net-zero CO_2_ emissions by 2060 through reducing its emissions at a constant rate of 2.5% annually. It is noteworthy to highlight that while the 1.5°C trajectory and the CN trajectory both follow the same socioeconomic pathway, they diverge in terms of carbon pricing (Table S3) and climate targets (Table S11), with the latter being more ambitious as it aims to emit less carbon during the study years. Under these scenarios, we project the future production and consumption landscapes of hydrogen fuel in China and the associated requirement for key infrastructure in Fig. 1.

The amount of hydrogen-fuel production in China would increase from 0.18 Mt/year in 2020 to 20.04, 32.05, and 38.00 Mt/year in 2060, under the BAU, 1.5°C, and CN scenario, respectively (Fig. 1A). Under the BAU scenario, the share of hydrogen fuel from coal and gas in the production mix would decline from a dominant 81% in 2020 to 46% in 2060, or 12.71 and 5.80% under the 1.5°C and CN scenarios, respectively, owing to the stringent requirements on achieving carbon mitigation targets. Greener hydrogen production technologies, which generate hydrogen with renewable energy such as bioenergy, and solar and wind power, would need to expand significantly faster under the 1.5°C and CN scenarios. Notably, solar- and wind-powered electrolytic hydrogen production would rapidly expand before 2050 in all three scenarios, incentivized by its sharply declining production cost thanks to technological progress (Table S4). Despite that the cost of biohydrogen with carbon capture and storage (BHCCS) remains higher than electrolytic hydrogen production technologies, the sky-high carbon price in those deep-decarbonization scenarios enables wide adoption of BHCCS. Thus, biomass as negative emission technology will witness a prompt increase in the hydrogen production mix from 2050 to 2060 in the 1.5°C [55.73%, 17.86 exajoules (EJ)] and CN (82.00%, 31.16 EJ) scenario.

On the consumption side, hydrogen for use as freight transport fuel would expand dramatically from a tiny share of 0.04% in 2020 to a dominant share in 2060 under all of the three scenarios. This is based on the projection that the cost of fuel cell electric vehicles (FCEVs) is going to decline rapidly to reach the cost parity with battery electric vehicles in a decade (see cost assumption of different technologies in Table S5). By contrast, the percentage of hydrogen for use as industrial energy would rapidly decline in the same period from 95.71% to 6.72, 27.59, and 37.61% in the three respective scenarios. Hydrogen for passenger transport such as bus and light-duty vehicles (LDVs) would constitute a small part of the hydrogen consumption mix, as shown in the right panel of Fig. 1A, with their share slightly decreasing from 5.15% under the BAU scenario to 3.93% under the CN scenario.

### Emerging infrastructure to support China’s hydrogen future

We map the future required deployment of key infrastructure, including hydrogen-fuel production equipment, industry fuel cells, FCEVs, and hydrogen refueling stations (HRSs), along the hydrogen value chain under the designed scenarios in Fig. 1B. Our results show that the installed capacity of hydrogen-fuel production will increase rapidly from the current 0.08 GW to 42.5, 62.2, and 69.1 GW under the BAU, 1.5°C, and CN scenario by 2040. To support this booming hydrogen-fuel production, there are three main sources, requiring different infrastructures. Firstly, technologies without CCS (carbon capture and storage) include biomass, coal chemical, natural gas steam reforming, and thermal splitting, whose installed capacity would maintain an increasing trend, reaching 46.2, 12.6, and 12.4 GW under the BAU, 1.5°C, and CN scenario by 2050. However, when the carbon price grows to sky-high, these technologies will become less competitive than the other two types of low even negative carbon emissions technologies. The second category of hydrogen production technologies is electrolysis, which offers versatile options for clean hydrogen production and exhibits high adaptability to renewable energy sources. Specifically, it can draw its power from renewable solar and wind energy, as well as directly from electricity, utilizing both central and forecourt methods. Our analysis indicates a significant growth in its installed capacity under the 1.5°C and CN scenarios, with a notable decrease by 2060 reflecting the challenges associated with deep decarbonization. Thirdly, technologies equipped with CCS include “biomass + CCS”, “coal chemical + CCS” and “NG steam reforming + CCS”, and those would not be deployed under the BAU scenario for their high cost. Under the 1.5°C and CN scenarios, the installed capacity of those would reach 104 and 163 GW in 2060, making up for 53.3 and 79.0% of the total installed capacity of hydrogen-fuel production, respectively.

In the hydrogen application side, hydrogen is typically converted by fuel cells to be used in transportation sector and in industries as heat or/and power, excluding its use as feedstock. In the industrial sector, the installed fuel cell capacity is poised to undergo remarkable expansion, commencing from virtually naught and advancing to 4.8, 12.3, and 15.9 GW by the year 2040, and 7.6, 50.1, and 81.0 GW by 2060 for the BAU, 1.5°C, and CN scenarios, respectively. In terms of fuel cells for transportation, the number of FCEVs is projected to grow 2.6–2.8 times between 2040 and 2060. As a benchmark, in 2020, there were approximately 7,300 FCEVs on the road, primarily trucks (59%) and buses (41%), with a smaller number being passenger vehicles (42). Our projections show that by 2040, there will be 0.8, 0.9, and 2.3 million LDVs; 15.1, 17.4, and 17.8 million trucks; and 1.9, 2.4, and 2.7 million buses for the BAU, 1.5°C, and CN scenarios, respectively. This surging demand for FCEVs will consequently drive up the need for hydrogen fueling infrastructure. Our estimates indicate a need for 25.8 thousand HRSs by 2040 under the CN scenario, scaling up to 80.6 thousand by 2060. To put this in perspective, as of 2022, a mere 358 HRSs existed. This underscores the significant infrastructure expansion required—increasing the number of HRSs by a staggering factor of over 200 times in the next four decades—to facilitate the carbon-neutral transition.

### Booming Pt demand with costly and risky supply

**Fig. 2.**
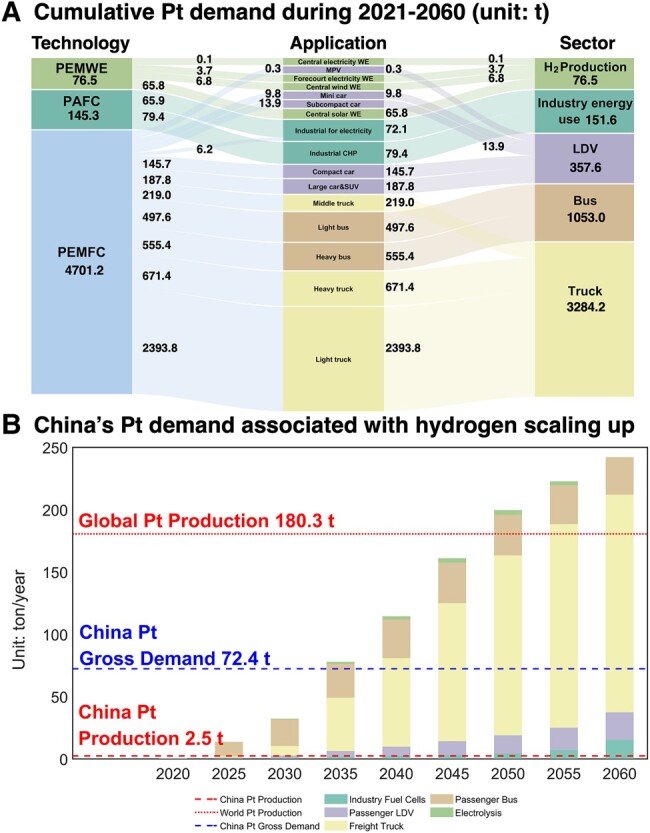
China's hydrogen development would overload the global platinum supply chain from 2021 to 2060 under the CN scenario unless supply growth is ensured by joint efforts and innovations to match the pace of demand growth. A) China's cumulative Pt demand structure from 2021 to 2060. B) China's Pt demand trend associated with the hydrogen energy development. Results in other scenarios can be found in Figs. S1 and S2. The following acronyms are used in A. PAFC, phosphoric acid fuel cell; PEMFC, proton exchange membrane fuel cell; PEMWE, polymer electrolyte membrane water electrolysis; MPV, multipurpose vehicle. LDV, light-duty vehicles. In B China's Pt primary production is the average from 2015 to 2020; China's current Pt gross demand and Global Pt primary production are the averages from 2013 to 2020.

We further linked the Pt demand to the corresponding hydrogen infrastructure along its value chain under the CN scenario in Fig. 2A (for other scenarios, see Supplementary material). The polymer electrolyte membrane (PEM) electrolyzer, employing Pt as a catalyst, would require 76.5 tons Pt during the whole period from 2021 to 2060, accounting for 1.6% of the cumulative demand (4,923 tons). Hydrogen energy consumption in the industrial sector would require 145.3 tons Pt, accounting for 3.0% of the cumulative Pt demand. In this sector, the phosphoric acid fuel cell would account for 32.3% of the total installed capacity of industrial fuel cells but would be responsible for a substantial 95.9% of the Pt consumption. In contrast, the proton exchange membrane fuel cell, while accounting for 26.5% of the installed capacity, would consume only 4.1% of the sector's total Pt demand. Compared with industrial fuel cells, the transportation sector would consume much large amount of Pt to convert energy through fuel cells, which would potentially account for 95.5% of the cumulative Pt demand (4,923 tons). Among those, the fuel cell passenger vehicles would use only 8.0% of the transportation Pt demand because of their comparative disadvantage in cost-effectiveness per passenger. Fuel cell buses would be the sector with the highest demand for platinum until 2030 due to policy and public support that would promote the expansion of this technology. After 2030, as technology matures, the fleets of FCEV trucks and the associated Pt demand would gradually increase, as a consequence, FCEV trucks would consume 3284.2t Pt cumulatively in the next 40 years, replacing buses as the leading Pt demand sector.

Figure 2B shows China’s annual trends of Pt demand by applications under the CN scenario (for other scenarios, see supplementary material). It is expected that China’s Pt demand associated with hydrogen would increase from 0.6 tons per year (tons/year) in 2020 to 242 tons/year in 2060 (about 3.3 times China’s current Pt gross demand of 72.4 tons/year) in all final uses. In the coming decade, China’s hydrogen Pt demand is expected to increase rapidly to 32 tons/year, which is 13 times China’s current production capacity. This would intensify China’s dependence on overseas Pt supply. After 2030, hydrogen’s Pt annual demand would increase at an accelerated rate [9.5% compound annual growth rate (CAGR) during 2030–2050], surpassing China’s current gross demand of Pt by 2035 and outstripping the current global Pt production capacity (187.6 tons) by 2050. If the annual Pt production capacity were sustained, China would need to cumulatively acquire 4,826 tons Pt from international market over 40 years. It means that China must expand its oversea platinum supply capacity to ensure a timely delivery of hydrogen infrastructure.

The growing large Pt demand will pose China’s hydrogen development at high risks. At present and foreseen future, platinum is of high concentration at both the company level and the country level makes global platinum supply more vulnerable to the risks caused by escalating trade tensions, changing political conditions, rising protectionism, social turmoil, and natural disasters (43, 44). For instance, in 2019, 92% of global virgin platinum was supplied by only 5 companies, and 95% was sourced from 3 countries (South Africa, Russia, and Zimbabwe), with over 70% of global Pt supply coming from a single mining deposit, namely the Bushveld igneous complex platinum deposit (19–21). The intensifying geopolitical conflicts, labor disputes, and safety stoppages are becoming greater forces to take a notable toll on current annual supply (45). Aside from physical resource supply and other constrains such as environmental and social impacts (20), the price fluctuation of Pt in the international market is another major concern. As Pt is a legitimate means of investment, the higher demand of Pt will cause higher price, which would translate into higher capital gain and may in turn attract more hot money to hoard up Pt inventory, thus driving up such hydrogen development costs (26). This becomes severer for China without own supply sources. Under the stable price assumption, the total monetary cost of China’s cumulative Pt import is estimated to reach 165 billion USD, implying 4.24 billion USD per annum on average, which would significantly raise the cost burdens for China to reach CN pledge. The speculative market might amplify the market shocks, further aggravating China’s Pt cost. Thus, national stockpile of Pt is highly recommended for China, while the timing and the Pt scale of which should be carefully selected.

### Importance of Pt circular economy strategies

**Fig. 3.**
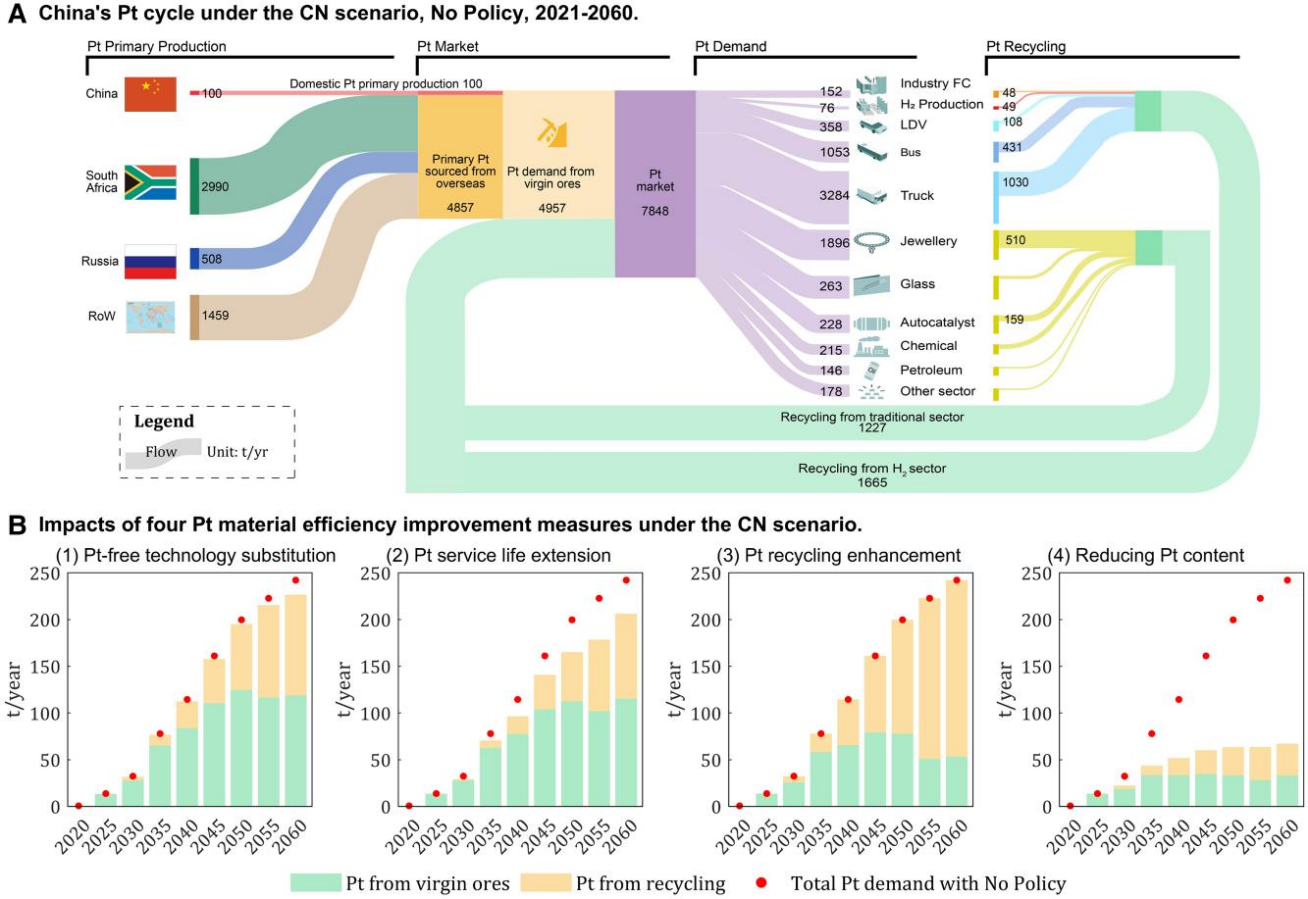
China’s platinum supply and consumption mix from 2021 to 2060 under the CN scenario A), and the impacts of four Pt material efficiency improvement measures on the Pt demand associated with hydrogen fuel B). The four measures are: (1) Pt-free technology substitution; (2) Pt service life extension; (3) Pt recycling enhancement; and (4) reducing Pt content. Results in other scenarios can be found in Figs. S3–S6.

Material efficiency improvement strategies such as increasing recycling efficiency, product redesign and material substitution can improve Pt supply resilience in the face of future uncertainties (46–48). Here, we quantified the potential benefits of four sets of material-based Pt efficiency strategies over Pt life cycle, including: (1) Pt-free technology substitution; (2) Pt service life extension; (3) Pt recycling enhancement (Tables S6 and S7); and (4) reducing Pt content. The detailed information on these four sets of strategies can be found in Table S8. In addition to Pt used in hydrogen value chain, we further incorporate Pt usage in traditional applications (e.g. autocatalysts; Tables S16–S18 and Fig. S12) into our Pt cycle framework (Fig. S11 and Table S15). Figure 3A presents the overview of China’s future Pt cycle across the global supply chain with accumulated flows from 2020 to 2060 (illustrated using the CN scenario as an example). The potential impacts of these strategy settings on future Pt demand and the associated supply sources are summarized in Fig. 3B.

The Pt material saving potential of adopting Pt-free technology to a maximum extent (strategy set 1) alone is quite limited, being only 140 tons, which translates to a savings of only 3% when compared to the no policy (NP) scenario (without adopting any circular economy strategy). This limitation arises because Pt-free technologies, such as SOFC and MCFC, are primarily employed within the industrial fuel cell sector and have little to no influence in other sectors. In the absence of the other three strategy sets, the total cumulative Pt demand would reach 4,783 tons. The Pt saving potential of adopting Pt service life extension (strategy set 2) alone is moderately higher at 15%. This often-overlooked set of technologies could curb the future Pt virgin source demand to around 111 tons/year from 2045 to 2060, and it can curtail the total cumulative Pt demand to 4,183 tons. The adoption of Pt recycling enhancement (strategy set 3) alone may not help to reduce the direct Pt demand, but curb the demand for Pt virgin resources which would peak at 104 tons in 2048, followed by a gradual decrease to nearly 53 tons in 2056–2060. Furthermore, it can be expected that nearly half of Pt demand can be met through Pt recycling since 2050. If the recyclable Pt from conventional vehicles is considered (49–51), a higher volume of recycling Pt can be expected.

In sharp contrast to sets (1)–(3), the sole adoption of reducing Pt content (set 4) yields a remarkable 62% reduction in the cumulative Pt demand over the entire study period, diminishing it from the baseline of 4,923 tons as defined by the NP scenario to a mere 1,855 tons. This strategy stands out as the most effective means of alleviating the platinum supply pressure. In terms of annual demand, it steadfastly maintains at nearly 65 tons over 2046–2060. Regarding the Pt demand from virgin ore sources, it peaks at about 39 tons in 2046–2050, subsequently receding to 29 tons during 2051–2060.

To summarize, over the next 40 years, proactive adoption of these four sets of measures could potentially enable China to reduce its import of Pt by a magnitude ranging from 103 to 2126 tons, saving 4–73 billion USD. Nevertheless, considering Pt application in traditional sectors, China would still need to import a substantial 4097–4857 tons from overseas until 2060. This quantity translates to 63–75 times of China’s domestic reserves, emphasizing the prevailing severity of the platinum supply risk.

## Discussion

At the global level, hydrogen is expected to meet 10–24% of global final energy needs by 2050 to comply with the Paris Agreement (52, 53). Such an unprecedented growth of hydrogen, as a key energy carrier, can greatly reshape the international energy geopolitics, resulting in new dependencies and rivalries between nations (2, 54–56). The existing literature has focused on factors such as operational costs (55), and the access to renewables and water of hydrogen development (56, 57). There is a lack of attention to the national opportunities, competitiveness, and key challenges related to the mineral base of hydrogen economy, which our study can help to address. Unlike the previous work from IEA (28) and others (29–31), our analysis cannot only project future demand of Pt but also provide a more solution-orientated analysis toward such platinum–hydrogen conflicts: Firstly, our integrated assessment model can provide a deep-dive analysis of the Pt demand in different infrastructure along hydrogen value chains with consideration of different technologies and different climate targets. Secondly, our MFA-assisted supply-chain analysis, beyond the demand-side projection, can cover the whole life cycle of Pt to identify the potential bottlenecks and feasible solutions. Here, we provide a comprehensive analysis of strategies ranging from Pt-free technology substitution, Pt service life extension, Pt recycling enhancement, to reducing Pt content, the impact and effectiveness of which on closing demand–supply gap is quantified to guide stakeholders to implement corresponding strategies.

Nevertheless, our analysis argues the Pt supply is still highly risky despite the implementation of those domestic strategies. Our supply-chain analysis (Fig. 3A) ascertains that if the current import structure (Fig. S10) persist, China would need to import 2,990 tons of Pt from South Africa, which represents approximately 9.2% of South Africa’s Pt reserves. Additionally, 508 tons would need to be sourced from Russia, which translates to roughly 24.4% of Russia’s Pt reserves. In those nations, any socioeconomic and political instabilities in and around may disrupt Pt supply chain and greatly alter hydrogen progress. Fortunately, Southern Africa has kept a credible track record with regard to expanding Pt mine output to meet the rising demand. For example, the policy in Europe that incentivized low CO_2_ emitting diesel vehicles has led to a supply growth from under 65 tons to over 160 tons in South Africa (19). On the other hand, the long-term impact of the ongoing Russia–Ukraine war would make the global Pt supply even more concentrated at other sources in South Africa and Zimbabwe. For example, London Platinum and Palladium Market, the world's biggest metals trade hub, has removed Russian refiners from its list of “good delivery” refiners (58). Assisted by our full supply-chain analysis, we argue the well-designed long-term cooperation between major hydrogen energy producers and platinum producers for responsible sourcing, trading, processing of hydrogen and platinum are highly needed. Thus, China needs to monitor and improve its diplomatic and industrial relations with those nations to fulfill its carbon-neutral pledge.

Global Pt sourcing will be more challenging for China, as the developed nations raise their hydrogen ambitions too. US Department of Energy releases Hydrogen Energy Project Plan, proposing an overall strategic framework for hydrogen energy research, development and demonstration over the next 10 years (59). European Commission establishes EU Clean Hydrogen Energy Alliance, announcing the roadmap for EU’s hydrogen energy development until 2050 (60). Japan announces the Basic Hydrogen Strategy, shifting hydrogen energy from a “hydrogen-based society” concept to a country’s basic strategy (61). These hydrogen ambitions rely on the effective operation of Pt supply chain (62, 63). Thus, a better understanding of the competition dynamics in global Pt market would help design effective measures to prevent potential geopolitical conflicts, which can be explored through our approach. Amid this escalating competition for Pt, nations must formulate and solidify effective hydrogen policies that extend beyond mere general principles and brief guidelines. Once such policies are put in place and become trusted by industrialists, uncertainty on future platinum demand will be significantly reduced and global platinum supply will be far more likely to grow in the coming decades. Aside from the competition for Pt, the hydrogen pathways may also be limited by other scarcer Platinum Group Metals, particularly the iridium (Ir). According to our analysis, in the absence of any reduction in Ir loadings, a significant proportion (over 50% after 2045 in CN scenario) of global Ir mining output (7 tons/a) would be necessitated solely to cater to China’s burgeoning polymer electrolyte membrane water electrolysis (PEMWE) market demand (Figs. S13 and S14).

There are additional factors which may add to the potential supply bottlenecks, such as declining ore grade (20, 64, 65) and the property mined as a by-product (21). An example of the former is that the mines in South Africa have experienced declining ore grade throughout their lifetime (5), which requires deeper digging; and an example of the latter is that palladium and platinum are mined as coproducts of nickel in Russia. Land disturbance triggered by the low grade of Pt (66) consequently results in additional environmental cost, entailing higher governance cost and stricter capacity permits. Moreover, given the fact that the timeframe to open a new mine typically last for 1–2 decades (67), how to scale-up Pt supply chains far ahead of the emergence of a new hydrogen economy geared up by large-scale green-hydrogen production, transportation, and millions of fuel cell vehicles remains a great challenge.

All these additional factors can also add volatilities to the already extremely volatile Pt market. For example, there was an electricity outage in South Africa which caused a drop of about 3% in annual platinum production, lead to a 40% platinum price hike within weeks (26). Our explorative analysis on price sensitivity shows that Pt price would increase by up to 15 times in the CN scenario, driven by the rapidly growing Pt demand (Table S9). In the extreme case that supply does not response to higher price and keeps unchanged, by 2060, the resultant Pt price hiking could result in platinum comprising as much as 81% of the cost share for industrial hydrogen-fuel technology (Fig. S7), implying that the Pt price in this extreme case could be the determining factor to the cost competitiveness of hydrogen-fuel technologies. This urges a systemic analysis on the feedback of Pt price to hydrogen cost competitiveness, which is informative for stakeholders to prepare adaptive strategies. For example, while PEMWE for producing green hydrogen using renewable electricity are the preferred technology, very high platinum costs would not limit the production of green hydrogen as the alternative technology of alkaline electrolyzers could fill the gap.

Securing a resilient Pt supply chain to support China's hydrogen ambition presents a considerable challenge. Fortunately, our model indicates that the significant increase in Pt demand is likely 10–20 years away from now, providing a time window for strategic planning in Pt supply capacities. Furthermore, the notion of a circular Pt economy holds promise. Since Pt-containing devices possess a higher Pt grade than virgin ores, the advancements in Pt recovery technology could pave the way for a more sustainable Pt economy. This would not only boost Pt yields but also simultaneously mitigate the environmental impact associated with its extraction and processing (68).

Echoing prior research (69), resource constraints significantly challenge the shift toward global low-carbon energy, essential for mitigating climate change. Our framework integrates climate change ambition with hydrogen energy and platinum supply, leading to informed platinum management strategies amid widespread uncertainties. This analysis emphasizes the need for global policymakers to harmonize the pursuit of low-carbon initiatives with access to critical minerals and highlights the importance of international cooperation. By adopting circular economy strategies, uncertainties regarding resource availability can be reduced, contributing to a balanced path for effective and sustainable climate action.

## Materials and methods

### Integrated assessment model

We used GCAM (version 5.2) to investigate hydrogen production and consumption structure in China’s carbon-neutral energy system due to its richness and extensibility in technological representation of hydrogen-related technologies. GCAM is a bottom-up, technology-rich integrated assessment model that depicts key interactions across economic, energy, land, water, and climate systems. GCAM divides the world into 32 regions, with China being one of these 32 regions. The China module covers three energy consumption sectors (industry, building, and transport). For hydrogen technologies, GCAM covers seven types of production-side technologies (coal, natural gas, nuclear, solar, wind, grid electricity, and biomass), five types of consumption-side applications, including industrial hydrogen use (power generation and cogeneration), transport hydrogen use (freight transport, passenger bus, and passenger LDVs). More information about the model can be openly accessed via the following link: https://github.com/JGCRI/gcam-core/releases (70).

In this study, these sectoral energy demands are projected considering economic growth and population changes in line with the Shared Socioeconomic Pathway 2 (SSP2 (71)). These demands are satisfied by a variety of final energies, which involve more than 100 energy technologies that have different costs and CO_2_ emission factors. GCAM solves for the market price of hydrogen energy so that supplies and demands of hydrogen by each technology are balanced. Detailed assumptions related to each category of hydrogen technology are presented in Tables S12 and S13. To be noted, hydrogen is treated exclusively as an energy stock in GCAM, thereby, the use of hydrogen as a chemical feedstock in industries like steel manufacturing is ignored. Detailed documents about hydrogen energy in GCAM can be found at http://jgcri.github.io/gcam-doc/v5.2/toc.html.

### Scenario description

We explored hydrogen energy use and infrastructure demand in China’s energy system under three different climate targets (Table S3). (i) The BAU scenario, where no climate target constraint is posed to the economy. (ii) The 1.5 warming target (1.5°C) scenario, under which China’s emission mitigation pathway is aligned with the goal of the Paris Agreement-limiting temperature rising by the end of 21st century to 1.5°C compared to preindustrial level. (iii) CN scenario, under which China’s CO_2_ emissions will be mitigated by a constant annual rate of 2.5% over 2020–2060 and reach net-zero emissions in 2060. The emission trajectories of different scenarios are described in Table S10 and Table S11.

### Hydrogen supply-chain infrastructure

Integrating the Integrated Assessment Model (IAM) with a stock-driven dynamic MFA model, we are able to estimate infrastructure demand of hydrogen energy industry. The evolution of market shares for different subtechnologies from 2020 to 2060 is depicted in Fig. S8, while changes in Pt intensity of hydrogen technologies over the same period are illustrated in Fig. S9. When analyzing Pt demand for hydrogen infrastructure, historic data used to evaluate Pt supply and demand are summarized in Table S14.

## Supplementary Material

pgae172_Supplementary_Data

## Data Availability

All data are included in the manuscript and/or Supplementary material.

## Preprints

This manuscript was posted on a preprint: http://dx.doi.org/10.2139/ssrn.4329451.
