# Supplementary material for: Incorporating platinum circular economy into China’s hydrogen pathways toward carbon neutrality
Source: PNAS Nexus. 2024 May 14;3(5):pgae172. doi: 10.1093/pnasnexus/pgae172 (PMC11093126; doi:10.1093/pnasnexus/pgae172)
Supplement: pgae172_Supplementary_Data [file pgae172_supplementary_data.docx]

**
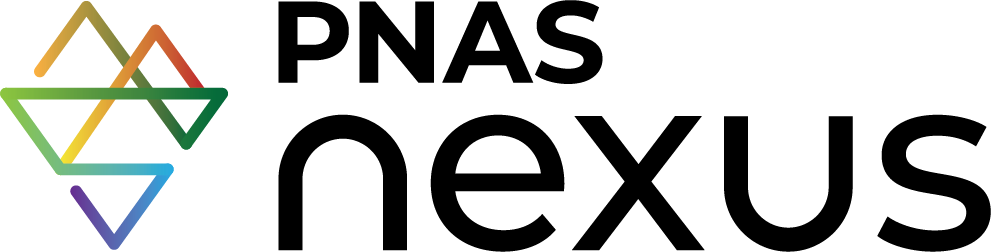
**

**Supplementary Information for**

**Incorporating Platinum Circular Economy into China's Hydrogen Pathways towards Carbon Neutrality**

Peng Wang ^1,5#^, Chenyang Wang ^1,7#^, Jiashuo Li ^2^, Klaus Hubacek ^3^, Laixiang Sun ^4^, Fan Yang ^2,6*^, Kuishuang Feng ^4, *^ & Wei-Qiang Chen ^1,5*^

^1^ Key Lab of Urban Environment and Health, Institute of Urban Environment, Chinese Academy of Sciences, Xiamen 361021, China

^2^ Institute of Blue and Green Development, Shandong University, Weihai 264209, China

^3^ Integrated Research on Energy, Environment and Society (IREES), Energy and Sustainability Research Institute Groningen, University of Groningen, Groningen 9747 AG, the Netherlands

^4^ Department of Geographical Sciences, University of Maryland, College Park, MD 20742, USA

^5^ University of Chinese Academy of Sciences, Beijing, 101408, China

^6^ Department of Planning, Aalborg University, Aalborg 9000, Denmark

^7^ Robert M. Buchan Department of Mining, Queen's University, Kingston, K7L 3N6, Canada

^#^ These authors contributed equally to this work: P.W., C. W.

* Corresponding authors:

fanyang@plan.aau.dk (F. Y.), [kfeng@umd.edu](mailto:kfeng@umd.edu) (K.F.), [wqchen@iue.ac.cn](mailto:wqchen@iue.ac.cn) (W.-Q. C.)

**This PDF file includes:**

Supplementary text

Figures S1 to S17

Tables S1 to S19

SI References

## *Supplemental Methodology Details*

The relationship between theoretical outflows, inflows and in-use stock of hydrogen energy infrastructure can be expressed as follows.

$F_{{Outflow}_{t}}^{tech}=\sum_{t=2015}^{t=T} F_{{Inflow}_{t}}^{tech}\times P_{t}^{T}$ (1)

$F_{{Inflow}_{t}}^{tech}=F_{{Stock}_{t}}^{tech}-F_{{Stock}_{t-1}}^{tech}+F_{{Outflow}_{t}}^{tech}$ (2)

$F_{{Outflow}_{t}}^{tech}$ refers to the capacity of hydrogen energy technology, tech, that has reached the end of their service life in year t; $F_{{Inflow}_{t}}^{tech}$ refers to the newly added capacity of hydrogen energy technology, tech, in year $t$; $P_{t}^{T}$ represents the probability of infrastructure being deployed in year t and retired in year $T$, which is given by assuming normal distribution, $N(\mu,\sigma^{2})$, for infrastructure lifetime. According to GCAM's default settings, we set 7 years for the average lifetime $(\mu)$ of the solar, wind, and grid electrolysers, 40 years for nuclear, biomass, and natural gas power plants, 10 years for that of industrial fuel cells (both power generation and cogeneration) and 15 years for fuel cell vehicles. The standard deviation $(\sigma)$ is set as one-fourth of the lifetime; $F_{{Stock}_{t}}$ and $F_{{Stock}_{t-1}}$ refer to in-use stock of technology in year t and $t-1$, respectively.

The in-use capacity of a technology $F_{{Stock}_{t}}^{tech}$ is estimated based on the hydrogen energy $E_{t}^{tech}$produced or consumed in the contemporary year $t$, with assumed capacity factor ${CF}_{t}^{tech}$ (detailed assumptions of capacity factor for each technology are in Table S12 and S13).

$F_{{Stock}_{t}}^{tech}=$ $E_{t}^{tech}\times{CF}_{t}^{tech}$ (3)

Pt demand of hydrogen infrastructure: Pt demand of hydrogen technology in year t is calculated as follows:

$D=\sum_{t} \sum_{tech} D_{t}^{tech}$ (4)

$D_{t}^{tech}=$ $F_{{inflow}_{t}}^{tech}\times\sum_{subtech}^{tech} {MS}_{t}^{subtech}\times{PI}_{t}^{subtech}$ (5)

where D is total Pt demand of hydrogen technology, $D_{t}^{tech}$ is Pt demand of hydrogen technology, tech, in year t. $F_{{inflow}_{t}}^{tech}$ is the newly-added capacity of technology, $tech$, in year $t$;${MS}_{t}^{subtech}$ stands for the market shares of different sub-technology categories for technology, tech. ${PI}_{t}^{subtech}$ ispolish Pt intensity of the sub-technology, $subtech$, in year $t$. Historic data used for evaluating Pt supply and demand are summarised in Table S14.

Market shares of different sub-technologies are determined based on market data and analysis. For hydrogen electrolysis, PEM (polymer electrolyte membrane) electrolysers is well suited for integration with intermittent renewable energies. Therefore, we assumed that PEM electrolysers takes a 100% share of the electrolysers market, and in fact, market share of PEMWE with a 5% reduction can only cause a 0.08%-0.10% reduction on the total Pt demand, see Supplemental Sensitivity Analysis. For hydrogen transport use, PEMFC is currently the only commercialised engine technology for fuel cell electric vehicles. It is thus assumed to take all the market share of transport hydrogen use. For industrial hydrogen use, four sub-technologies exist, namely, PEMFC (polymer electrolyte membrane fuel cell), PAFC (phosphoric acid fuel cell), SOFC (solid oxide fuel cell), and MCFC (molten carbonate fuel cell). The current market share of fuel cells for industrial and industrial CHP are collected from the E4tech report [1]. The evolution of market share during 2020-2060 can be viewed in Figure S8.

Pt intensities of those hydrogen technologies are collected from extensive literature review [2–6]. The evolution of Pt intensity of PEM electrolyser was collected from NREL [7]. For PEMFC, its Pt intensity during 2020-2060 is assumed to follow the trajectory by Ministry of Industry and Information Technology [8–10]. For PAFC, its Pt intensity was 19 times that of PEMFC in the same year [11,12]. This study assumed the same ratio between Pt intensity of PAFC and that of PEMFC throughout the study period. Detailed data on the evolution of Pt intensity of hydrogen technologies are in Figure S9. For PEMFC, we select the current advance technology as the base case, and design Pt loading reduction pathways for evaluating the pressure of future supply chain. For PAFC and PEM WE, Pt loading with a 5% reduction can only result in a 0.04% - 0.15% reduction on the total Pt demand, see Supplemental Sensitivity Analysis.

Pt price and its cost share in hydrogen technologies: To estimate Pt price, we first established the relation between price and demand through a regression model, following the approach proposed by Sun et al [13]. A logistic relationship between Pt price and demand was assumed as follows:

$\frac{P-P_{L}}{P_{U}-P_{L}}=\frac{1}{1+e^{-c_{1}-c_{2}*D}}$ (6)

where, $P$ is the price for Pt; $D$ is Pt demand. Those are historical data from 1975 to 2019 [14–17]. $P_{L}$ and $P_{U}$ are the assumed lowest and highest price limits. The lower limit of Pt price, $P_{L}$, is assumed according to historical data of Pt price. The upper limit of Pt price, $P_{U}$, is estimated by applying the Hotelling's rule [18] and assuming different expected rates of return. The Hotelling's rule defines that the optimal pricing strategy to maximise social economic rent of a non-renewable resource is by increasing the price of the resource at a rate equals to the expected rate of return. Miners might set those Pt mineral rents at a lower price in a competitive market to fulfill their individual interests, leaving social welfare at stake. Thereby, Hotelling's price can be viewed as an upper limit. The rule can be expressed as:

$\frac{P_{U}(t+1)}{P_{U}(t)}=1+\theta$ (7)

where $P_{U}(t)$ is the upper bound of unit price as time $t$; $\theta$ is the expected rate of return. The expected rate of return can spread widely with varying social-economic statues [19]. We therefore developed three scenarios to reflect its intrinsic uncertainty. 1) A low rate of return, $\theta=1.4\%$, which reflect the social discount rate assumed in Stern's report[20]; 2) A moderate rate, $\theta=5.4\%$, reflecting Nordhaus's assumption of the social discount rate; and 3) a high rate, $\theta=8\%$, aligning with the empirical evidence shown in South African investments [21].

The logistic model gives the best estimate of $c_{1}$ and $c_{2}$. It can then be used to estimate the evolution of Pt price based on future total Pt demand. To do this, we need first to build up a baseline estimate of total Pt demand other than those from the expansion of hydrogen-fuel industry, and then add Pt demand from the expansion of the hydrogen-fuel industry to the baseline estimate. Annual Pt demand has been stably increasing. To project future Pt demand, we simply fit a straight line using historical demand data. Pt demands from the expansion of hydrogen-fuel industry with various technologies under different climate and cost scenarios were then added up to the baseline future Pt demand estimate.

The cost share of Pt in different hydrogen technology is calculated as follows:

$Pt cost share in technology i= \frac{Pt price\times Pt intensity of i}{Non-fuel cost of technology i}$ (8)

where Pt prices and Pt intensities are given above; non-fuel costs of hydrogen technology including initial capital investment and subsequent non-fuel operational costs, which are derived from GCAM.

## *Supplemental Tables*

Table S1 Comparison of our hydrogen demand forecast with previous studies

| Reference | H_2_  demand | Region | Climate target | H_2_  application | End-use sector coverage |
| --- | --- | --- | --- | --- | --- |
| Hydrogen council[22] | 200 Mt  in 2050 | China | 1.5℃ | H_2_ as fuels and H_2_ for industrial applications | Building (20%) Industry (50%) Power (10%) Transport (40%) |
| International Energy Agency[23] | 90 Mt  in 2060 | China | Carbon neutrality | H_2_ fuels and H_2_ for industrial applications | Industry (40%) Refinery (20%) Transport (25%) Others (15%) |
| China hydrogen alliance[24] | 130 Mt  in 2060 | China | Carbon neutrality | H_2_ as fuels and H_2_ for industrial applications | Building (5.5%) Industry (60%) Power (4.5%) Transport (30%) |
| Shell[25] | 25.5 Mt  in 2060 | China | 2℃ | H_2_ as fuels | Heavy industry (25%) Transport (22%) Others (53%) |
| BP[26] | 93 Mt  in 2050 | China | 1.5℃ | H_2_ as fuels and H_2_ for industrial applications | Not Available |
| Deloitte[27] | 139 Mt in 2050 | China | Carbon neutrality | H_2_ as fuels and H_2_ for industrial applications | Not Available |
| CN scenario, this study | 20.0 Mt  in 2060 | China | Carbon neutrality | H_2_ as fuels | Industry (37.6%) Transport (62.4%) |
| 1.5℃ scenario, this study | 32.1 Mt  in 2060 | China | 1.5℃ | H_2_ as fuels | Industry (27.6%) Transport (72.4%) |
| BAU scenario, this study | 38.0 Mt  in 2060 | China | No climate target | H_2_ as fuels | Industry (6.7%) Transport (93.3%) |

Note: In comparing these tables, please note differences in time, scenarios, underlying assumptions, technology level assumption, and the hydrogen application area.

Table S2 Review on the key Pt demand results from previous studies

| Reference | Pt demand* | Technology  Scope | Key assumption | Key results |
| --- | --- | --- | --- | --- |
| (Raymond, Sterck and Clifford, 2022)[28] | 31.1 t (1M oz) **p.a.** by 2030 124.4t (4M oz) **p.a**. by 2040  40.4t (1.3M oz) **p.a**. by 2028 208.4t (6.7M oz) **p.a.** by 2040 | FCEVs | **Policy-driven scenario:** FCEV adoption is driven by government and regional subsidies, incentives, and legislated targets. FCEVs reaches 2m in 2030 and 11m in 2040. **Commercially enhanced adoption scenario:** government and regional policies have engendered infrastructure critical mass. FCEVs reaches 3m in 2030 and 15m in 2040. | Supply without increases can cause platinum scarcity, which would hinder FCEV growth rates, while Pt production can be expanded with time to satisfy the demand growth. |
| (Tong et al., 2022)[29] | nearly 280-350 t **p.a.** in 2050 | Transportation (FCEVs, ICEVs) | **Energy scenario:** carbon neutrality within half a century. **Technology assumption:** S1: Technological Substitution–substituting ICEs with FCVs and BEVs.  S2: Diversified niches-considering the path-dependency of technological change. S3: Landscape change–combined with a systematic change in mobility landscape | There is an imbalance between supply and demand for Pt between regions, calling for a global coordination to enhance the material efficiency for Pt |
| (Hao et al., 2019)[10] | 33.8 kt(D1) 93.2 kt(D2) 56.1 kt(D3)  2010-2100 **cumulatively** | Transportation (FCEVs, ICEVs) | **Population and economic growth:**  SSP2.  **Vehicle sales, scrappage, and stock:** the global vehicle sales will increase from the current level of 97 million to 144 million in 2030 (49% increase), 192 million in 2050 (98% increase), and 220 million in 2100 (127% increase). **Technology assumption:** D1: FCVs penetrating only the HDV segment. D2: FCVs into the HDV & LDV segment. D3: Lowering PGM loading of fuel cells. | PGMs are not likely to be a constraint for the mass deployment of FCEVs at the global level, there could be significant supply risks due to resource location. |
| (Alonso, Field and Kirchain, 2012)[30] | 59 kt  2010-2050 **cumulatively** | All platinum-using industries | **Energy scenario:** IEA projected fleet for meeting 450 greenhouse gas goals. **Technology assumption:** simulate historical platinum usage and its growth rate for individual Pt-using industries, and FCEVs reach 12% in 2035. | The demand can only be met by the primary supply curve （projected based on 5% exponential growth rate）. |
| (Rasmussen et al., 2019)[31] | 51.4 kt (the most aggressive fuel cell scenario)  2016-2050 **cumulatively** | All platinum-using industries | **FCEVS:** bottom-up approach, fuel cell rapid development scenario **Electrolyser:** extrapolate Danish to all other countries  **Other sectors:** assumption based on their historical patterns | Only in high demand scenarios and when fuel cell market penetration is higher than the expected, the aggregate demand to 2050 will exceed the 2016 global platinum reserves. |
| (Elshkaki, 2013)[32] | over 1.4 kt **p.a.** in 2050 over 8.0 kt **p.a.** in 2100 | All platinum-using industries | **Fuel Cells** are modelled based on the demand for vehicles, the share of Fuel Cell Vehicles (FCVs). FCEVs market share reaches 50% by the year 2050 and 100% by the year 2100. **Catalytic Converters** are modelled based on the demand for vehicles, FCVs market share, CC market share. **Other Applications** are modelled based on socio-economic variables, metal price and time. | Platinum identified resources are expected to deplete with or without the introduction of FCVs before the end of the century. |

Note: Those values are obtained roughly based on their result presentations.

Table S3 Carbon price setting of each climate scenario
(Unit: $/t Carbon)

| Year | BAU | 1.5°C[18] | CN[33] |
| --- | --- | --- | --- |
| 2020 | 0.00 | 0 | 10.51 |
| 2025 | 0.00 | 0 | 163.096 |
| 2030 | 0.00 | 127.79 | 233.011 |
| 2035 | 0.00 | 163.095 | 269.54 |
| 2040 | 0.00 | 208.156 | 302.835 |
| 2045 | 0.00 | 265.665 | 342.229 |
| 2050 | 0.00 | 339.064 | 393.914 |
| 2055 | 0.00 | 432.741 | 473.921 |
| 2060 | 0.00 | 552.299 | 547.108 |

Table S4 Cost evolution of hydrogen production technology from 2020 to 2060 (Unit: $/GJ)

| **Technology** | **2020** | **2025** | **2030** | **2035** | **2040** | **2045** | **2050** | **2055** | **2060** |
| --- | --- | --- | --- | --- | --- | --- | --- | --- | --- |
| Biomass to H_2_ | 7.11 | 7.03 | 6.90 | 6.79 | 6.81 | 6.80 | 6.76 | 6.72 | 6.67 |
| Biomass to H_2_ CCS | 20988.10 | 20758.00 | 20803.40 | 20579.70 | 20360.80 | 20365.40 | 20153.30 | 19945.60 | 19995.10 |
| Coal chemical | 4.11 | 4.06 | 4.03 | 3.97 | 3.92 | 3.87 | 3.83 | 3.79 | 3.76 |
| Coal chemical CCS | 19170.60 | 18976.30 | 18992.30 | 18803.60 | 18618.70 | 18607.40 | 18428.20 | 18252.40 | 18245.60 |
| Electrolysis | 20.95 | 20.34 | 18.87 | 17.73 | 17.31 | 17.13 | 16.99 | 16.77 | 16.54 |
| Natural gas steam reforming | 3.57 | 3.66 | 3.63 | 3.66 | 3.76 | 3.85 | 3.99 | 4.08 | 4.10 |
| Natural gas steam reforming CCS | 8909.44 | 8828.80 | 8858.83 | 8779.89 | 8715.26 | 8732.77 | 8669.56 | 8594.82 | 8625.17 |
| Thermal splitting | 7.30 | 7.31 | 7.32 | 7.33 | 7.35 | 7.37 | 7.39 | 6.56 | 5.72 |
| Electricity+Electrolysis | 20.95 | 20.34 | 18.87 | 17.73 | 17.31 | 17.13 | 16.99 | 16.77 | 16.54 |
| Solar+Electrolysis | 7.40 | 6.42 | 4.94 | 3.83 | 3.46 | 3.37 | 3.27 | 3.18 | 3.09 |
| Wind+Electrolysis | 9.44 | 8.66 | 7.38 | 6.44 | 6.16 | 6.15 | 6.12 | 5.99 | 5.81 |
| Electricity+Electrolysis (Forecourt) | 24.83 | 24.11 | 22.57 | 21.31 | 20.77 | 20.48 | 20.23 | 19.89 | 19.57 |
| Natural gas steam reforming (Forecourt) | 13.91 | 14.13 | 13.84 | 13.77 | 13.95 | 14.06 | 14.31 | 14.33 | 14.04 |

Data Source: Reference [34,35]

Table S5 Cost evolution of different road transport technology from 2020 to 2060 (Unit: $/vehicle*km)

| **Sector** | **Scenario** | **Subsector** | **Technology** | **2020** | **2025** | **2030** | **2035** | **2040** | **2045** | **2050** | **2055** | **2060** |
| --- | --- | --- | --- | --- | --- | --- | --- | --- | --- | --- | --- | --- |
| LDV | BAU, 1.5°C &CN | Mini Car | BEV | 0.047 | 0.044 | 0.041 | 0.041 | 0.042 | 0.043 | 0.043 | 0.042 | 0.042 |
|  |  | Mini Car | FCEV | 0.055 | 0.052 | 0.047 | 0.048 | 0.049 | 0.049 | 0.048 | 0.047 | 0.047 |
|  |  | Subcompact Car | BEV | 0.070 | 0.065 | 0.058 | 0.060 | 0.061 | 0.062 | 0.062 | 0.061 | 0.061 |
|  |  | Subcompact Car | FCEV | 0.075 | 0.070 | 0.065 | 0.066 | 0.067 | 0.068 | 0.067 | 0.065 | 0.066 |
|  |  | Compact Car | BEV | 0.103 | 0.098 | 0.091 | 0.093 | 0.095 | 0.097 | 0.097 | 0.097 | 0.096 |
|  |  | Compact Car | FCEV | 0.109 | 0.103 | 0.096 | 0.098 | 0.099 | 0.101 | 0.100 | 0.099 | 0.099 |
|  |  | Multipurpose Vehicle | BEV | 0.207 | 0.206 | 0.205 | 0.204 | 0.203 | 0.202 | 0.198 | 0.194 | 0.190 |
|  |  | Multipurpose Vehicle | FCEV | 0.165 | 0.166 | 0.167 | 0.169 | 0.170 | 0.172 | 0.170 | 0.167 | 0.167 |
|  |  | Large Car& SUV | BEV | 0.154 | 0.142 | 0.130 | 0.129 | 0.128 | 0.127 | 0.127 | 0.126 | 0.126 |
|  |  | Large Car& SUV | FCEV | 0.157 | 0.148 | 0.136 | 0.134 | 0.132 | 0.133 | 0.132 | 0.130 | 0.131 |

| **Sector** | **Scenario** | **Subsector** | **Technology** | **2020** | **2025** | **2030** | **2035** | **2040** | **2045** | **2050** | **2055** | **2060** |
| --- | --- | --- | --- | --- | --- | --- | --- | --- | --- | --- | --- | --- |
| Truck | BAU, 1.5°C &CN | Heavy Truck | BEV | 0.081 | 0.073 | 0.063 | 0.057 | 0.052 | 0.046 | 0.046 | 0.044 | 0.044 |
|  |  | Heavy Truck | FCEV | 0.068 | 0.055 | 0.041 | 0.035 | 0.031 | 0.026 | 0.025 | 0.025 | 0.025 |
|  |  | Middle Truck | BEV | 0.113 | 0.102 | 0.088 | 0.080 | 0.073 | 0.065 | 0.065 | 0.063 | 0.062 |
|  |  | Middle Truck | FCEV | 0.099 | 0.079 | 0.058 | 0.051 | 0.045 | 0.038 | 0.037 | 0.037 | 0.037 |
|  |  | Light Truck | BEV | 0.980 | 0.865 | 0.761 | 0.695 | 0.632 | 0.568 | 0.558 | 0.543 | 0.537 |
|  |  | Light Truck | FCEV | 0.797 | 0.642 | 0.467 | 0.408 | 0.350 | 0.291 | 0.289 | 0.285 | 0.287 |

| **Sector** | **Scenario** | **Subsector** | **Technology** | **2020** | **2025** | **2030** | **2035** | **2040** | **2045** | **2050** | **2055** | **2060** |
| --- | --- | --- | --- | --- | --- | --- | --- | --- | --- | --- | --- | --- |
| Bus | BAU, 1.5°C &CN | Heavy Bus | BEV | 0.029 | 0.022 | 0.022 | 0.022 | 0.021 | 0.021 | 0.021 | 0.020 | 0.020 |
|  |  | Heavy Bus | FCEV | 0.029 | 0.021 | 0.017 | 0.016 | 0.015 | 0.015 | 0.014 | 0.014 | 0.014 |
|  |  | Light Bus | BEV | 0.051 | 0.039 | 0.038 | 0.037 | 0.037 | 0.036 | 0.036 | 0.036 | 0.036 |
|  |  | Light Bus | FCEV | 0.051 | 0.037 | 0.031 | 0.027 | 0.026 | 0.026 | 0.025 | 0.025 | 0.025 |

Data Source: [35,36]

Table S6 Recycling patterns for key hydrogen energy infrastructure

| **Sector** | **Subsector** | **Recycling Pattern** |
| --- | --- | --- |
| Industry use | H_2_ for industry energy use | Closed-Loop |
|  | H_2_ for industry CHP use |  |
| H_2_ Production-Electrolysis | H_2_ central production |  |
|  | H_2_ central production |  |
|  | H_2_ central production |  |
|  | H_2_ forecourt production | Open-Loop |
| LDV | Compact Car |  |
|  | Large Car and SUV |  |
|  | Mini Car |  |
|  | Multipurpose Vehicle |  |
|  | Subcompact Car |  |
| Truck | Light Truck |  |
|  | Middle Truck |  |
|  | Heavy Truck |  |
| Bus | Heavy Bus |  |
|  | Light Bus |  |

Table S7 Parament settings of different recycling patterns

| Recycling Indicator | General | | Enhancement | |
| --- | --- | --- | --- | --- |
|  | Closed  -loop | Open  -loop | Closed  -loop | Open  -loop |
| CR[37–39] | 100.0% | 70.0% | 100.0% | 99.0% |
| Recovery rate[40] | 78.0% | 78.0% | 95.0% | 95.0% |
| EOL-RR | 78.0% | 54.6% | 95.0% | 94.1% |

Note^31^:

1. CR is the abbreviation of old scrap collection rate, and describes the amount of the end-of -life metals which is collected and sent to the recycling chain.
2. Recovery rate is also known as recycling process efficiency rate, and describe the efficiency in the given recycling process.
3. EOL-RR is the abbreviation of end-of-life recycling rate, and refers to functional recycling, and includes recycling as a pure metal and as an alloy.
4. The relationship between these three variables are as follows:

EOL-RR = CR × Recovery rate.

Table S8 Description of circular economy scenarios

| Strategy | Technology market share^1^ | | Life time^2^ | | Recycling^3^ | | Material intensity^4^ | |
| --- | --- | --- | --- | --- | --- | --- | --- | --- |
|  | I | II | I | II | I | II | I | II |
| No policy | √ |  | √ |  | √ |  | √ |  |
| (1) Pt-free technology substitution^5^ |  | √ | √ |  | √ |  | √ |  |
| (2) Pt service life extension^6^ | √ |  |  | √ | √ |  | √ |  |
| (3) Pt recycling enhancement^7^ | √ |  | √ |  |  | √ | √ |  |
| (4) Pt basic reduction^8^ | √ |  | √ |  | √ |  |  | √ |

Note:

1. Technology market share: Type I means general technology mix; Type II means No-Pt technology mix. Please refer to Figure S8**.**
2. Life time: Type I means general lifetime, Type II means the extended lifetime. Please refer to Table S13**.**
3. Pt recycling enhancement: Type I adopts current recycling setting; Type II adopts enhanced recycling setting. Please refer to Table S6 and Table S7.
4. Material intensity: Type I means the current advanced intensity; Type II means the basic reduction intensity. Please refer to Figure S9.
5. Strategy (1) lies in the planning stage and aims to avoid Pt usage in H_2_ value chain by selecting alternative Pt-free technologies. For instance, in the industrial use of hydrogen energy, those emerging Pt-free technologies (e.g., SOFC and MCFC) are assumed to quickly replace PAFC and PEMFC and fully occupy the market by 2045.
6. Strategy (2) focuses on the in-use stage to extend key Pt technology service lifetime by ensuring asset durability and optimising operation.
7. Strategy (3) incorporates various end-of-life strategies for better management of end-of-life products and high efficiency of the Pt recovery process.
8. Strategy (4) complies with the principle of material reduction for the same hydrogen service, mainly through technology breakthroughs and R&D to reduce the platinum content in hydrogen equipment.

Table S9 Pt cost driven by the rapidly growing Pt demand under the CN scenario (Unit: $/g)

| Scenario | CM | | | CA | | | RR | | | AR | | |
| --- | --- | --- | --- | --- | --- | --- | --- | --- | --- | --- | --- | --- |
| Discount rate | D1 | D2 | D3 | D1 | D2 | D3 | D1 | D2 | D3 | D1 | D2 | D3 |
| 2021 | 48.60 | 68.12 | 70.91 | 48.56 | 67.89 | 70.64 | 48.56 | 67.88 | 70.63 | 48.54 | 67.77 | 70.50 |
| 2022 | 49.91 | 76.98 | 81.62 | 49.76 | 75.74 | 80.09 | 49.75 | 75.70 | 80.04 | 49.64 | 74.86 | 79.01 |
| 2023 | 50.79 | 84.95 | 91.72 | 50.50 | 82.12 | 88.08 | 50.49 | 82.03 | 87.96 | 50.26 | 79.84 | 85.19 |
| 2024 | 51.36 | 91.67 | 100.62 | 50.98 | 87.03 | 94.44 | 50.97 | 86.93 | 94.30 | 50.62 | 83.27 | 89.55 |
| 2025 | 51.73 | 96.92 | 107.83 | 51.28 | 90.61 | 99.19 | 51.28 | 90.61 | 99.19 | 50.89 | 86.07 | 93.18 |
| 2026 | 52.30 | 107.02 | 122.41 | 51.73 | 96.85 | 107.74 | 51.74 | 97.01 | 107.96 | 51.29 | 90.74 | 99.37 |
| 2027 | 52.87 | 121.44 | 145.06 | 52.30 | 106.90 | 122.24 | 52.20 | 104.97 | 119.38 | 51.67 | 96.01 | 106.56 |
| 2028 | 53.20 | 132.89 | 164.81 | 52.66 | 115.39 | 135.27 | 52.47 | 110.64 | 127.89 | 51.92 | 99.94 | 112.10 |
| 2029 | 53.37 | 141.09 | 180.07 | 52.90 | 122.10 | 146.15 | 52.64 | 114.79 | 134.33 | 52.11 | 103.33 | 116.98 |
| 2030 | 53.47 | 146.01 | 189.74 | 53.04 | 126.95 | 154.36 | 52.76 | 118.19 | 139.76 | 52.29 | 106.83 | 122.13 |
| 2031 | 53.74 | 165.28 | 231.79 | 53.37 | 140.80 | 179.53 | 53.07 | 128.04 | 156.24 | 52.57 | 113.13 | 131.73 |
| 2032 | 53.96 | 192.22 | 305.38 | 53.68 | 160.88 | 221.52 | 53.39 | 141.66 | 181.17 | 52.86 | 120.84 | 144.07 |
| 2033 | 54.04 | 208.11 | 360.31 | 53.83 | 174.82 | 255.56 | 53.55 | 151.01 | 199.97 | 53.04 | 126.98 | 154.40 |
| 2034 | 54.07 | 215.89 | 391.50 | 53.90 | 183.10 | 278.13 | 53.63 | 156.55 | 211.83 | 53.17 | 131.74 | 162.75 |
| 2035 | 54.07 | 217.49 | 398.34 | 53.92 | 186.29 | 287.33 | 53.66 | 158.75 | 216.71 | 53.25 | 135.34 | 169.26 |
| 2036 | 54.08 | 220.10 | 409.86 | 53.95 | 190.32 | 299.46 | 53.69 | 161.56 | 223.10 | 53.34 | 139.21 | 176.48 |
| 2037 | 54.10 | 226.94 | 442.09 | 53.99 | 198.13 | 324.61 | 53.75 | 166.80 | 235.42 | 53.43 | 144.00 | 185.74 |
| 2038 | 54.11 | 233.02 | 473.83 | 54.03 | 205.55 | 350.74 | 53.80 | 171.80 | 247.79 | 53.51 | 148.79 | 195.37 |
| 2039 | 54.12 | 238.35 | 504.35 | 54.05 | 212.48 | 377.46 | 53.85 | 176.56 | 260.13 | 53.59 | 153.59 | 205.42 |
| 2040 | 54.13 | 242.93 | 533.04 | 54.07 | 218.86 | 404.34 | 53.88 | 181.06 | 272.38 | 53.65 | 158.45 | 216.05 |
| 2041 | 54.13 | 247.12 | 561.61 | 54.09 | 225.04 | 432.82 | 53.92 | 185.61 | 285.34 | 53.72 | 163.45 | 227.47 |
| 2042 | 54.14 | 250.87 | 589.35 | 54.11 | 230.95 | 462.66 | 53.95 | 190.21 | 299.13 | 53.77 | 168.52 | 239.61 |
| 2043 | 54.14 | 253.93 | 613.76 | 54.12 | 236.21 | 491.73 | 53.97 | 194.63 | 313.05 | 53.82 | 173.55 | 252.26 |
| 2044 | 54.14 | 256.38 | 634.76 | 54.12 | 240.83 | 519.58 | 53.99 | 198.88 | 327.13 | 53.86 | 178.52 | 265.41 |
| 2045 | 54.14 | 258.32 | 652.39 | 54.13 | 244.84 | 545.76 | 54.01 | 202.98 | 341.39 | 53.90 | 183.42 | 279.02 |
| 2046 | 54.14 | 260.69 | 675.39 | 54.14 | 250.07 | 583.22 | 54.04 | 208.88 | 363.26 | 53.94 | 189.28 | 296.30 |
| 2047 | 54.14 | 262.26 | 691.72 | 54.14 | 254.23 | 616.26 | 54.06 | 214.55 | 385.89 | 53.97 | 195.09 | 314.54 |
| 2048 | 54.14 | 262.80 | 697.66 | 54.14 | 256.05 | 631.84 | 54.07 | 217.69 | 399.21 | 54.00 | 199.36 | 328.78 |
| 2049 | 54.14 | 262.81 | 697.83 | 54.14 | 256.37 | 634.66 | 54.07 | 218.78 | 403.99 | 54.01 | 202.23 | 338.75 |
| 2050 | 54.14 | 262.30 | 692.14 | 54.14 | 255.28 | 625.17 | 54.07 | 218.07 | 400.89 | 54.02 | 203.80 | 344.33 |
| 2051 | 54.14 | 261.65 | 685.28 | 54.14 | 254.08 | 615.03 | 54.07 | 217.74 | 399.45 | 54.03 | 205.43 | 350.29 |
| 2052 | 54.14 | 261.60 | 684.71 | 54.14 | 254.22 | 616.20 | 54.08 | 219.30 | 406.25 | 54.04 | 208.13 | 360.38 |
| 2053 | 54.14 | 261.73 | 686.13 | 54.14 | 254.73 | 620.49 | 54.08 | 221.35 | 415.51 | 54.05 | 211.04 | 371.67 |
| 2054 | 54.14 | 262.05 | 689.46 | 54.14 | 255.59 | 627.87 | 54.09 | 223.85 | 427.11 | 54.06 | 214.15 | 384.24 |
| 2055 | 54.14 | 262.49 | 694.24 | 54.14 | 256.74 | 637.96 | 54.10 | 226.71 | 440.97 | 54.07 | 217.43 | 398.10 |
| 2056 | 54.14 | 262.90 | 698.75 | 54.14 | 257.85 | 647.96 | 54.10 | 229.59 | 455.53 | 54.08 | 220.68 | 412.44 |
| 2057 | 54.14 | 263.18 | 701.96 | 54.14 | 258.68 | 655.70 | 54.11 | 232.18 | 469.23 | 54.09 | 223.68 | 426.31 |
| 2058 | 54.14 | 263.40 | 704.52 | 54.14 | 259.36 | 662.26 | 54.11 | 234.61 | 482.62 | 54.10 | 226.52 | 440.01 |
| 2059 | 54.14 | 263.58 | 706.53 | 54.14 | 259.93 | 667.74 | 54.12 | 236.87 | 495.61 | 54.10 | 229.17 | 453.39 |
| 2060 | 54.14 | 263.71 | 708.14 | 54.14 | 260.40 | 672.42 | 54.12 | 238.98 | 508.15 | 54.11 | 231.64 | 466.34 |

Note: D1=1.4; D2=5.4%; D3=8%.

Table S10 Description of climate scenarios

| Scenario | Social Economic Parament | Climate Target | Temperature Increase in 2060 |
| --- | --- | --- | --- |
| BAU | SSP2 | No climate policy constraints | 2.341°C |
| 1.5°C | SSP1 | No more than 1.5°C temperature increase in 2100 | 1.819°C |
| CN | SSP1 | Carbon emissions are reduced to zero in 2060 | 1.684°C |

Table S11 Carbon emissions pathway of each climate scenario
(Unit: Mt carbon/year)

| Year | BAU | 1.5°C | CN |
| --- | --- | --- | --- |
| 2020 | 3203.15 | 3207.16 | 3125.24 |
| 2025 | 3504.89 | 3510.71 | 2734.59 |
| 2030 | 3700.54 | 3707.34 | 2343.93 |
| 2035 | 3805.97 | 3813.61 | 1953.28 |
| 2040 | 3848.96 | 2709.80 | 1562.62 |
| 2045 | 3841.63 | 2063.31 | 1171.97 |
| 2050 | 3801.75 | 1257.86 | 781.31 |
| 2055 | 3702.83 | 616.80 | 390.66 |
| 2060 | 3595.11 | 172.94 | 0.00 |

Table S12 Classification and technical parameters of hydrogen production technology

| Technology | Type | Input | Plant Lifetime (year) | Stack Lifetime (year) | Capacity factor |
| --- | --- | --- | --- | --- | --- |
| Biomass to H_2_ | Central production | biomass | 40 | NA | 90% |
| Biomass to H_2_ CCS |  |  | 40 | NA | 90% |
| Coal chemical |  | coal | 30 | NA | 90% |
| Coal chemical CCS |  |  | 30 | NA | 90% |
| Electrolysis |  | electricity | 40 | 7 | 97% |
| Natural gas steam reforming |  | gas | 40 | NA | 90% |
| Natural gas steam reforming CCS |  |  | 40 | NA | 90% |
| Thermal splitting |  | nuclear | 40 | 7 | 90% |
| Electrolysis |  | solar | 40 | 7 | 50% |
| Electrolysis |  | wind | 40 | 7 | 50% |
| Electrolysis | Forecourt production | electricity | 20 | 7 | 86% |
| Natural gas steam reforming |  | gas | 20 | NA | 86% |

Note: More information about the model can be openly accessed via the following link <https://github.com/JGCRI/gcam-core/releases> [35].

Table S13 Lifetime setting of Pt-containing hydrogen equipment

| Sector | Subsector | General | | Extended | |
| --- | --- | --- | --- | --- | --- |
|  |  | Plant Lifetime (year) | Stack Lifetime (year) | Plant Lifetime (year) | Stack Lifetime (year) |
| Industry use | H_2_ for energy use | 20 | 10 | 20 | 15 |
|  | H_2_ for industry CHP use | 20 | 10 | 20 | 15 |
| LDV | Compact Car | 15 | 8 | 15 | 10 |
|  | Large Car and SUV | 15 | 8 | 15 | 10 |
|  | Mini Car | 15 | 8 | 15 | 10 |
|  | Multipurpose Vehicle | 15 | 8 | 15 | 10 |
|  | Subcompact Car | 15 | 8 | 15 | 10 |
| Truck | Light Truck | 15 | 8 | 15 | 10 |
|  | Middle Truck | 15 | 8 | 15 | 10 |
|  | Heavy Truck | 15 | 8 | 15 | 10 |
| Bus | Heavy Bus | 15 | 8 | 15 | 10 |
|  | Light Bus | 15 | 8 | 15 | 10 |
| H_2_ Production | Electrolysis | 20 | 7 | 20 | 10 |

Note: Extended stack life time are collected from literature[2,41].

Table S14 Data used for evaluating Pt demand

| **Variable** | **Value** | **Unit** | **Source** | **Process** |
| --- | --- | --- | --- | --- |
| China's Pt primary production | 2.5 | ton | BGS[42] | the average of data during 2015-2020 |
| Global Pt primary production | 180.3 | ton | USGS[43] | the average of data during 2013-2020 |
| China's current Pt gross demand | 72.4 | ton | SFA Oxford[44] | the average of data during 2013-2020 |
| China's recycling | 27.8 | ton | SFA Oxford[44] | the average of data during 2013-2020 |
| Pt content of PGM mines  (Pt ratio) | 51.3 | % | (Nassar, 2015)[37]  (Rasmussen et al., 2019)[31] | the average of 21 PGM mines |
| China's Pt reserve ^(1)^ | 65 | ton | China Mineral Resources[45] | Pt ratio * PGMs reserve of 2020 |
| Global Pt reserve^(1)^ | 35397 | ton | USGS[43] | Pt ratio * PGMs reserve of 2020 |
| Pt reserve of ZA | 32319 | ton | USGS[43] | Pt ratio * PGMs reserve of 2020 |
| Pt reserve of RUS | 2000.7 | ton | USGS[43] | Pt ratio * PGMs reserve of 2020 |
| Pt reserve of RoW | 1077.3 | ton | USGS[43] | Pt ratio * PGMs reserve of 2020 |
| Pt price ^(2)^ | 1064.6 | USD/troy ounce | LBMA[46] | the average of data during 2013-2021 |
| Pt import share of ZA ^(3)^ | 60.3 | % | UN Comtrade[47] | Share sourced from South Africa of the imported Pt |
| Pt import share of RUS ^(3)^ | 10.2 | % | UN Comtrade[47] | Share sourced from Russia of the imported Pt |
| Pt import share of RoW ^(3)^ | 29.4 | % | UN Comtrade[47] | Share sourced from RoW of the imported Pt |

Note: (1) The platinum reserves used in this study are based on data from 2020, and while they are relatively stable in the short term, they should not be directly cited for long-term predictions. As discussed by Rasmussen et al. (2019)[31], two methods for predicting future reserves have been identified, including considering the historical ratio of annual reserve change to production or forecasting future development based on historical growth rates. However, it is important to note that predictions of platinum reserves may not always be accurate or reliable due to the dynamic nature of the platinum industry and the potential impact of unexpected events or changes in the market such as natural disasters, accidents, geopolitical tensions, environmental regulations, changes in demand, or fluctuations in platinum prices.

(2) The figure is in 2020 dollars.

(3) The selected base year for our analysis is 2019 (Figure S10), aiming to represent the global platinum supply chain before the disruptive impact of the Covid-19 pandemic [48].

## *Supplemental Figures*


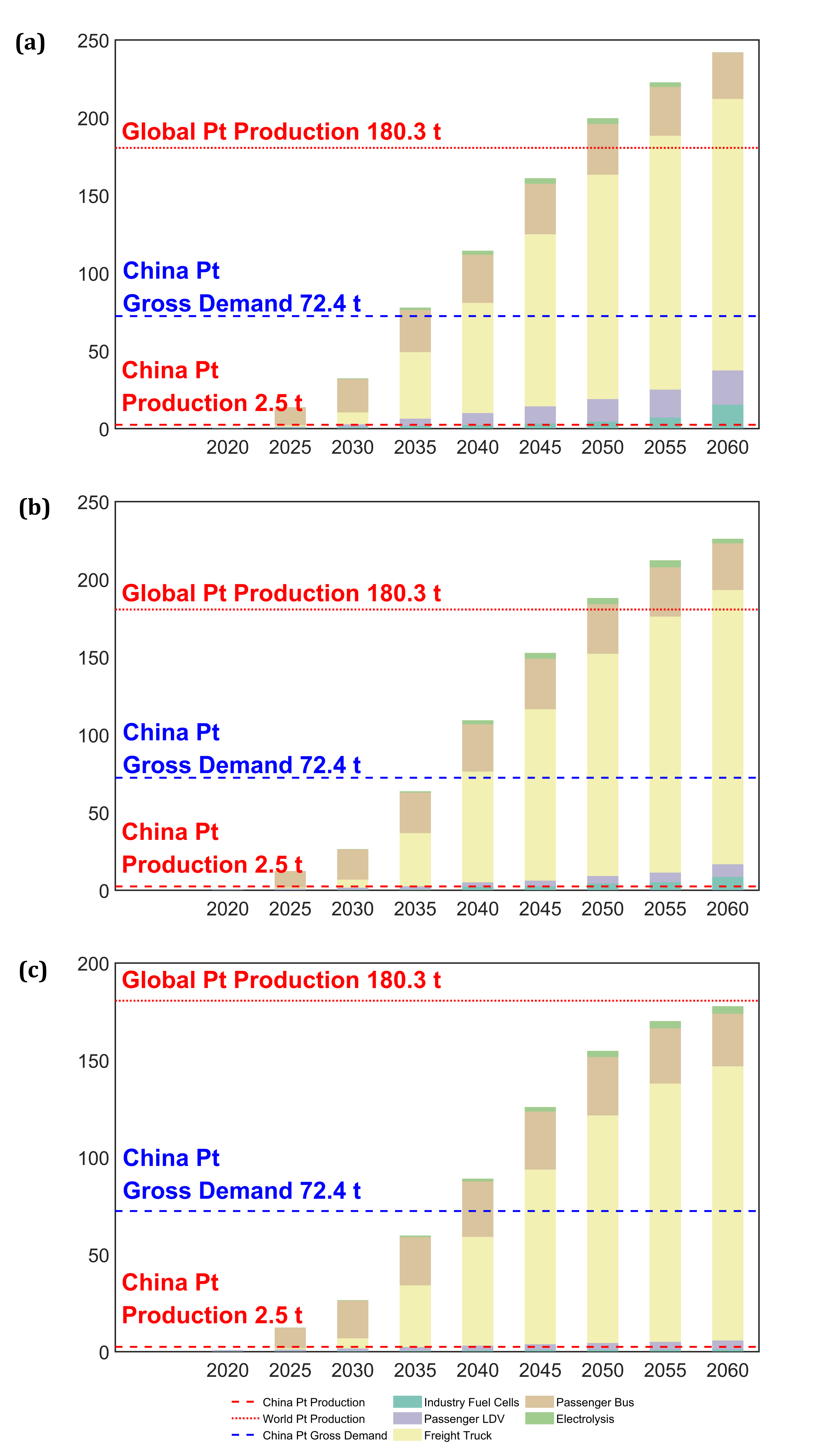


Figure S1 China's Pt demand trend associated with hydrogen development from 2021 to 2060 (unit: t). (a) the CN scenario, (b) the 1.5°C scenario, and (c) the BAU scenario. China's Pt primary production is the average from 2015 to 2020; China's current Pt gross demand and Global Pt primary production are the averages from 2013 to 2020.


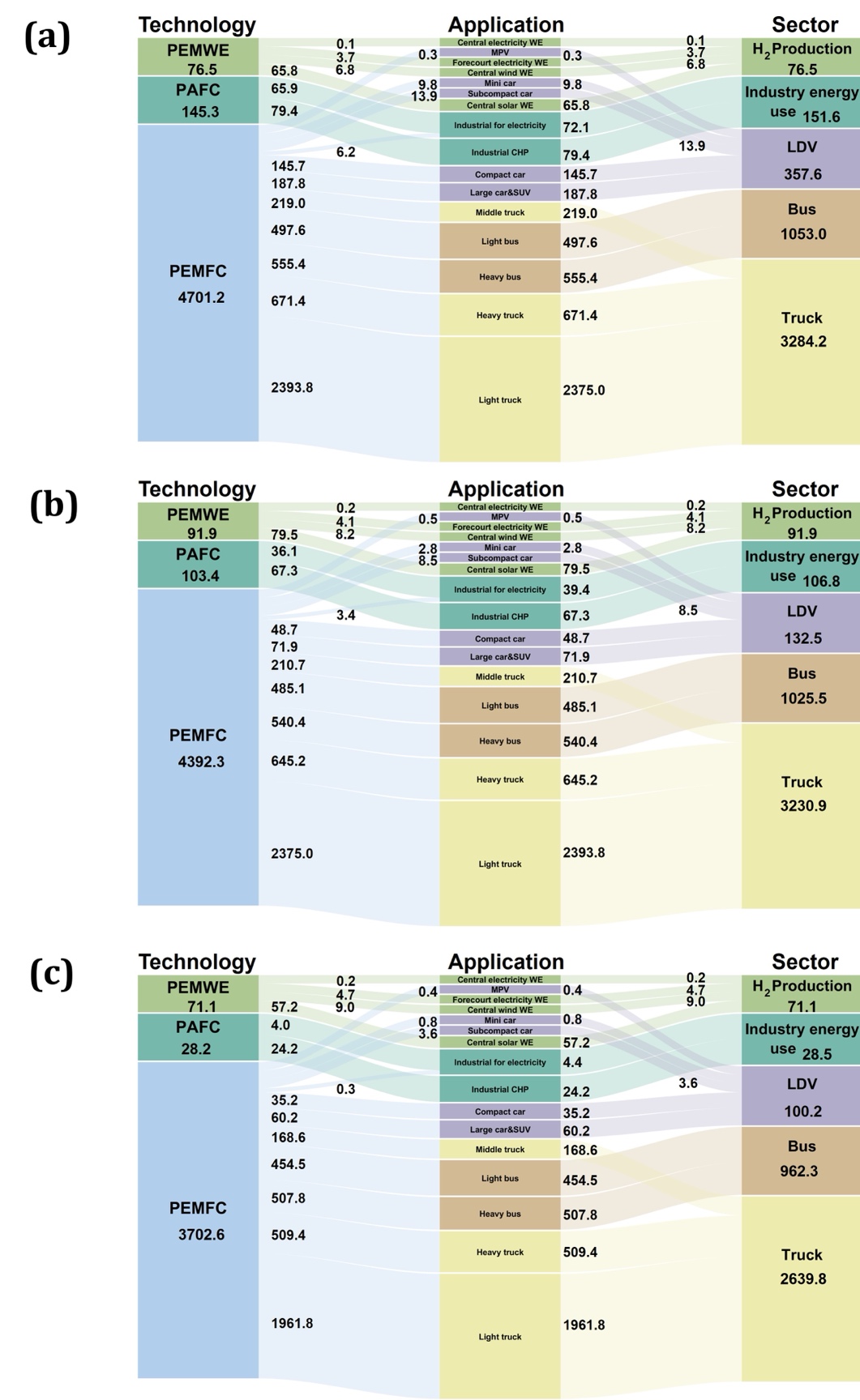


**Figure S2** China's cumulative Pt demand structure associated with hydrogen development during 2021-2060 (unit: t). (a) the CN scenario, (b) the 1.5°C scenario, and (c) the BAU scenario. The following acronyms are used. PAFC: Phosphoric Acid Fuel Cell; PEMFC: Proton Exchange Membrane Fuel Cell; PEMWE: Polymer Electrolyte Membrane Water Electrolysis; MPV: Multi-Purpose Vehicle; LDV: Light-duty Vehicles.


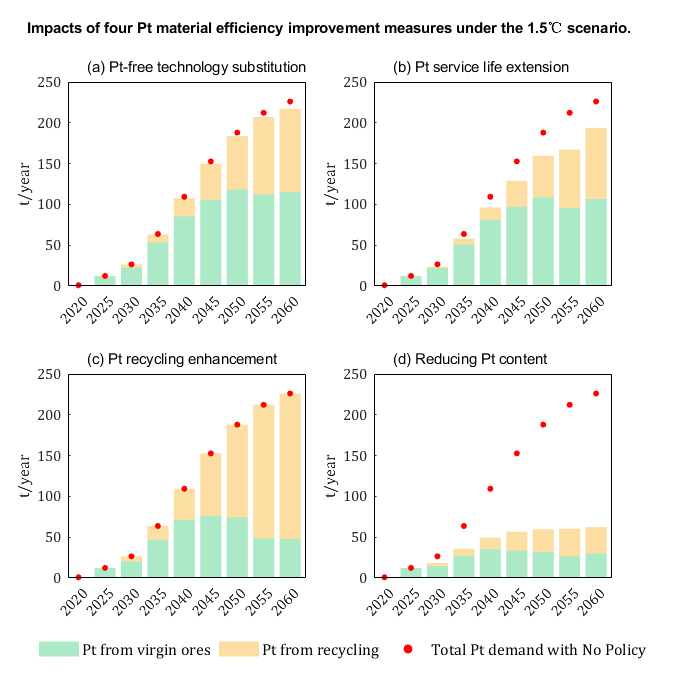


**Figure S3** Benefits of circular measures under the 1.5°C scenario. (a) Pt-free technology substitution; (b) Pt service life extension; (c) Pt recycling enhancement, and (d) Reducing Pt content.


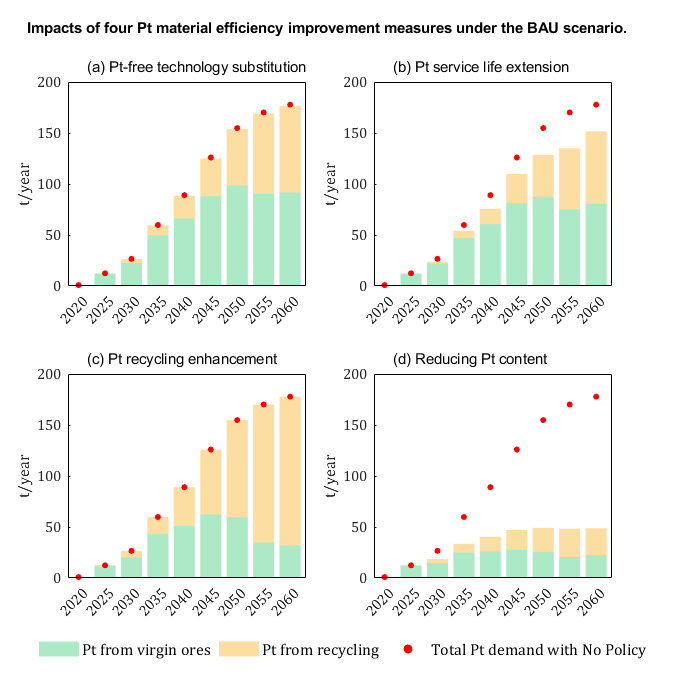


**Figure S4** Benefits of circular measures under the BAU scenario. (a) Pt-free technology substitution; (b) Pt service life extension; (c) Pt recycling enhancement, and (d) Reducing Pt content.


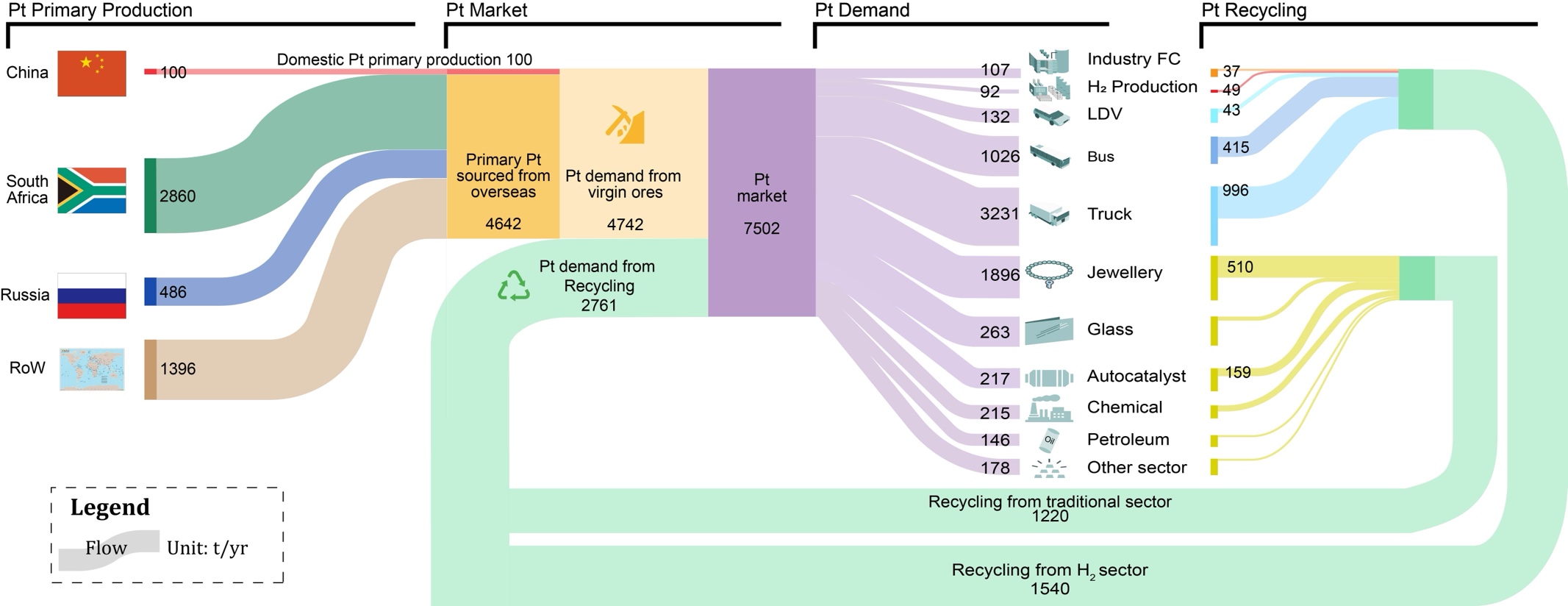


Figure S5 China’s platinum supply and consumption mix from 2021 to 2060 under the 1.5°C scenario


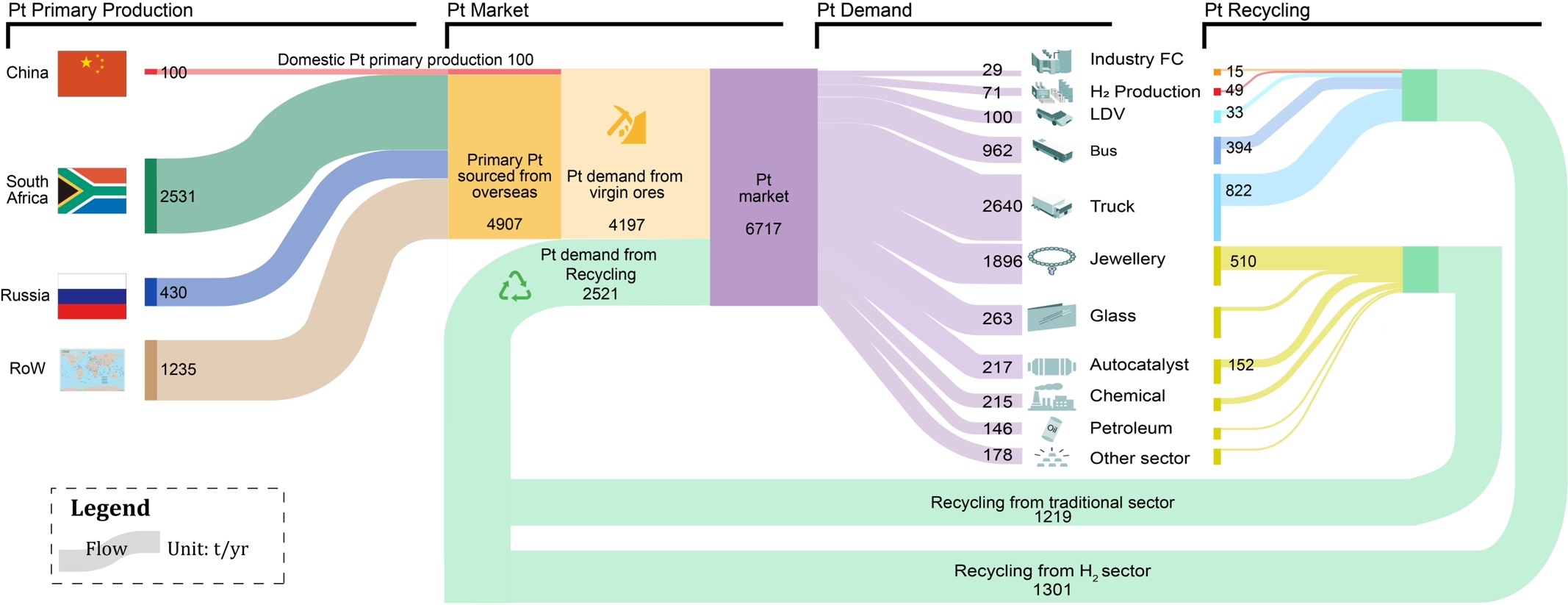


Figure S6 China’s platinum supply and consumption mix from 2021 to 2060 under the BAU scenario


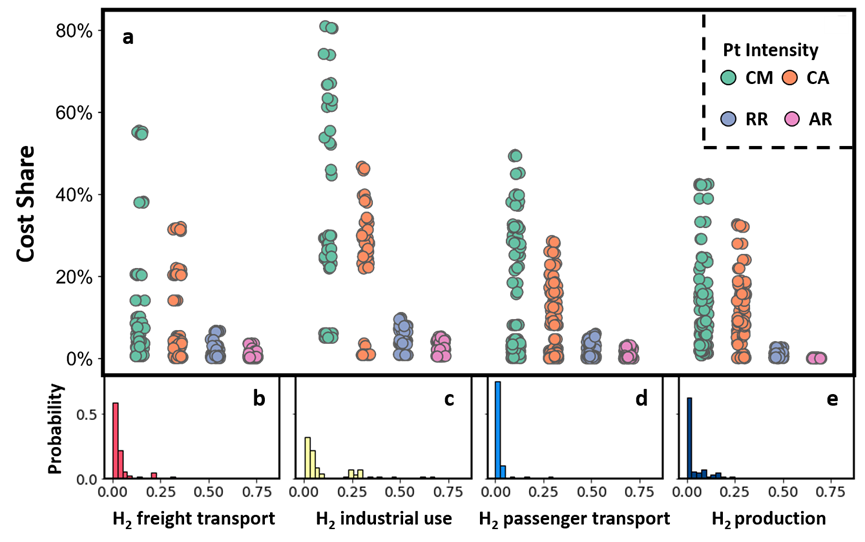


**Figure S7** Share of platinum cost in the total cost of individual hydrogen technologies in 2060. (a) Cost share of platinum in different hydrogen technologies with varying assumptions of platinum intensity (CM = Current Mainstream, CA = Current Advance, RR = Reference Reduction, AR = Advanced Reduction); (b) – (d) Probability distributions of platinum cost share in hydrogen technologies.


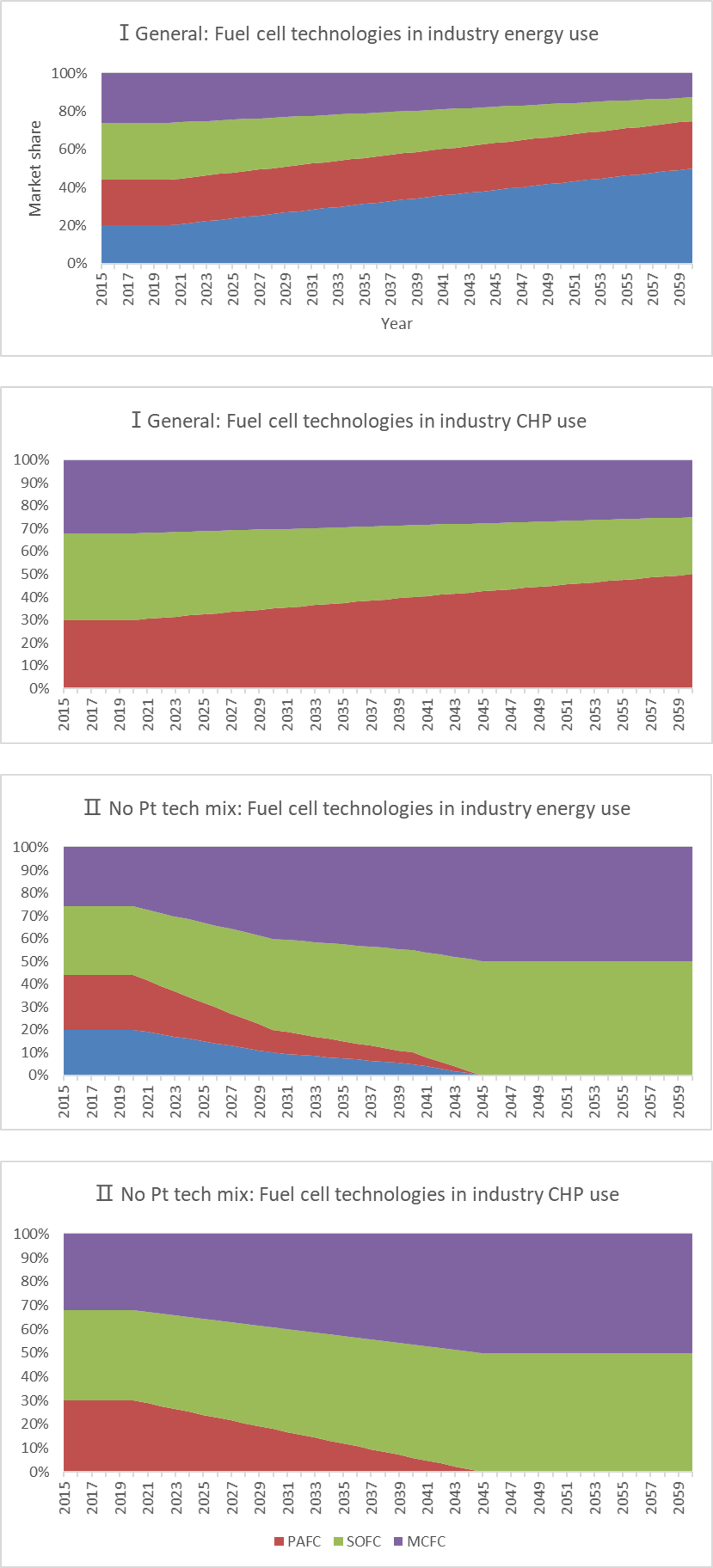


**Figure S8** Market share of fuel cell technologies in industry energy and CHP use. There is currently no statistic about the fuel cells type in industry energy and CHP use area. this study collects historical shipment and megawatts data of the fuel cells by type from E4Tech report[1], and provides a market share estimate worthy of reference in this area.
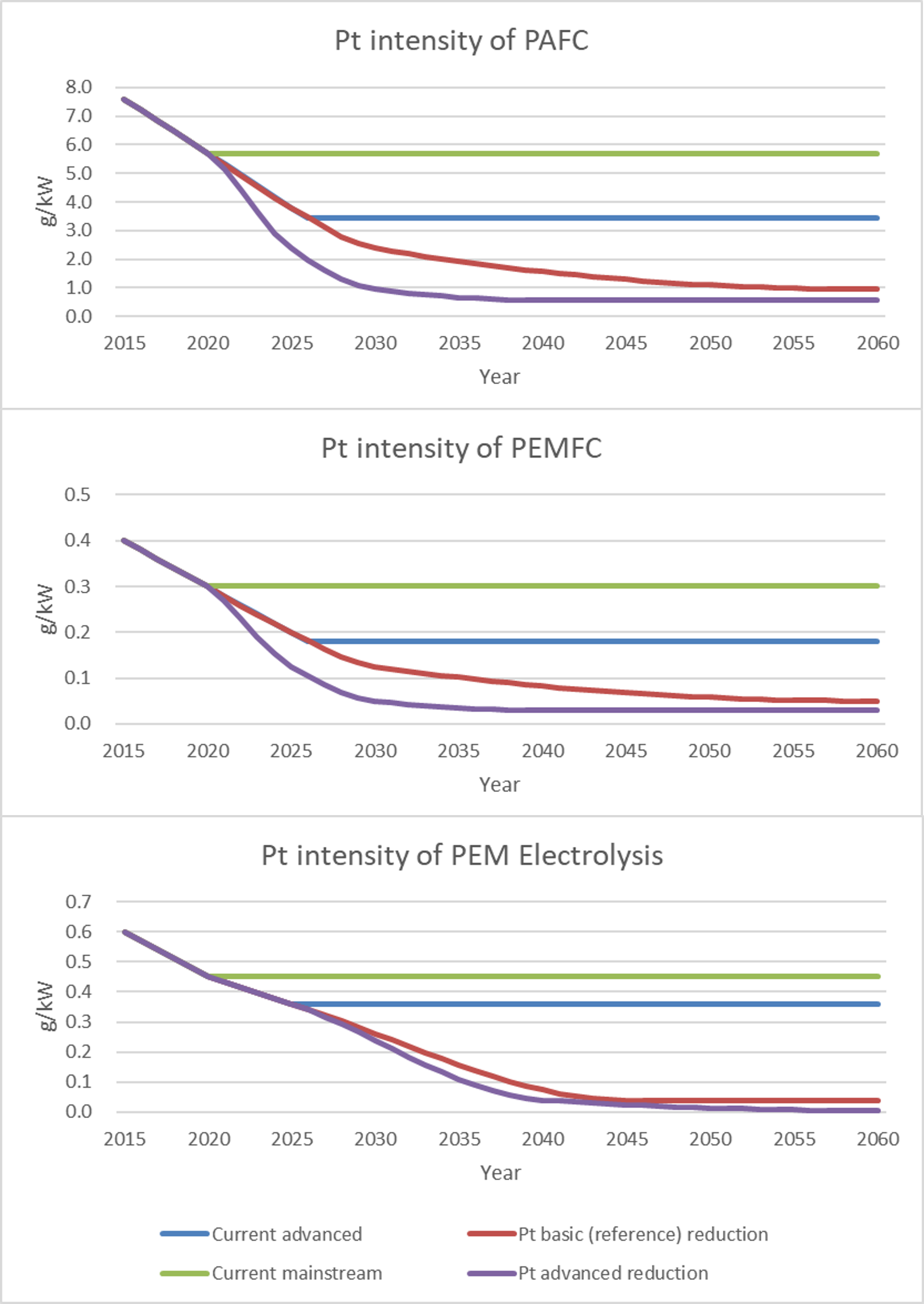


**Figure S9** Pt intensity evolution of key hydrogen technologies. Pt intensities of those hydrogen technologies are collected from extensive literature review[2–4,31,49]. The evolution of Pt intensity of PEM electrolyser was collected from NREL[7]. For PEMFC, its Pt intensity during 2020-2060 is assumed to follow the trajectory calibrated by Ministry of Industry and Information Technology of China[8–10]. For PAFC, its Pt intensity was 19 times that of PEMFC in the same year[11,12]. This study assumed the same ratio between Pt intensity of PAFC and that of PEMFC throughout the study period.


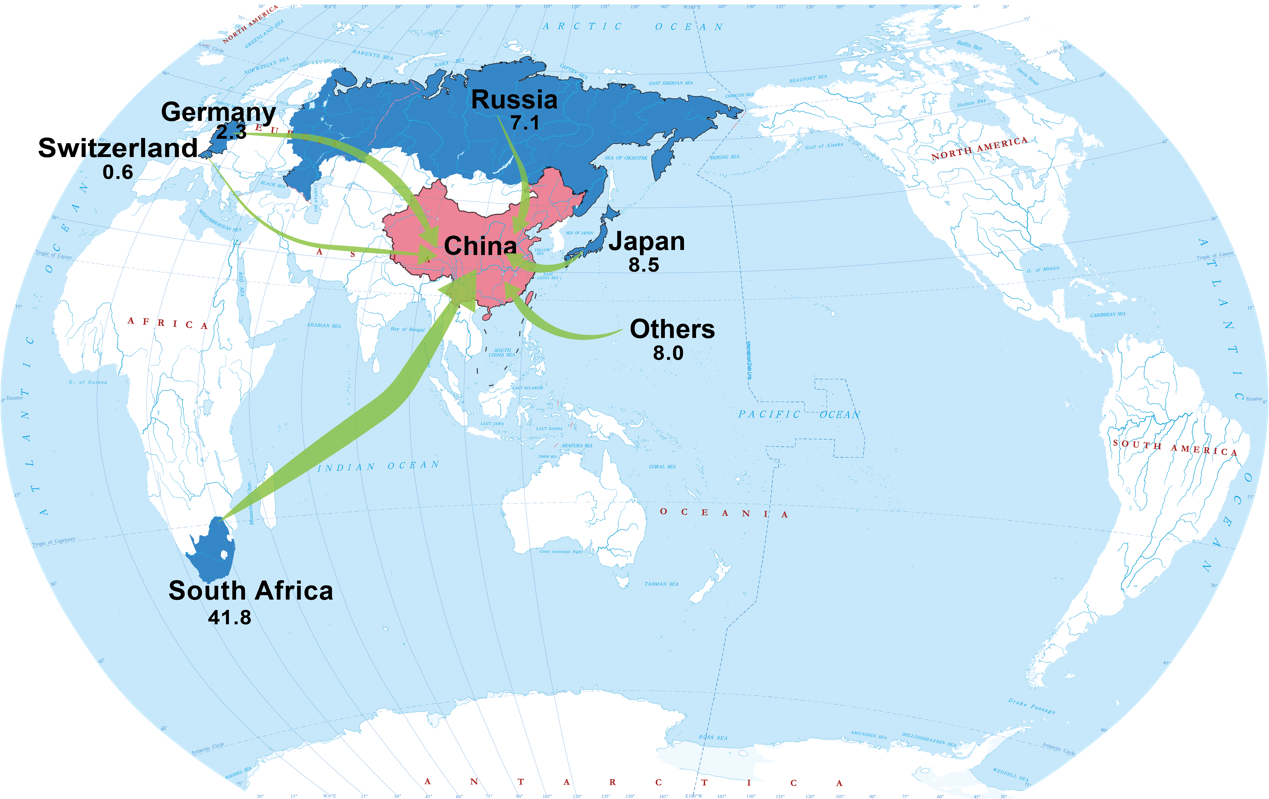


Figure S10 China’s Pt import map in 2019 (Unit: ton/year)

## *Supplemental explanation of Pt cycle in the future*


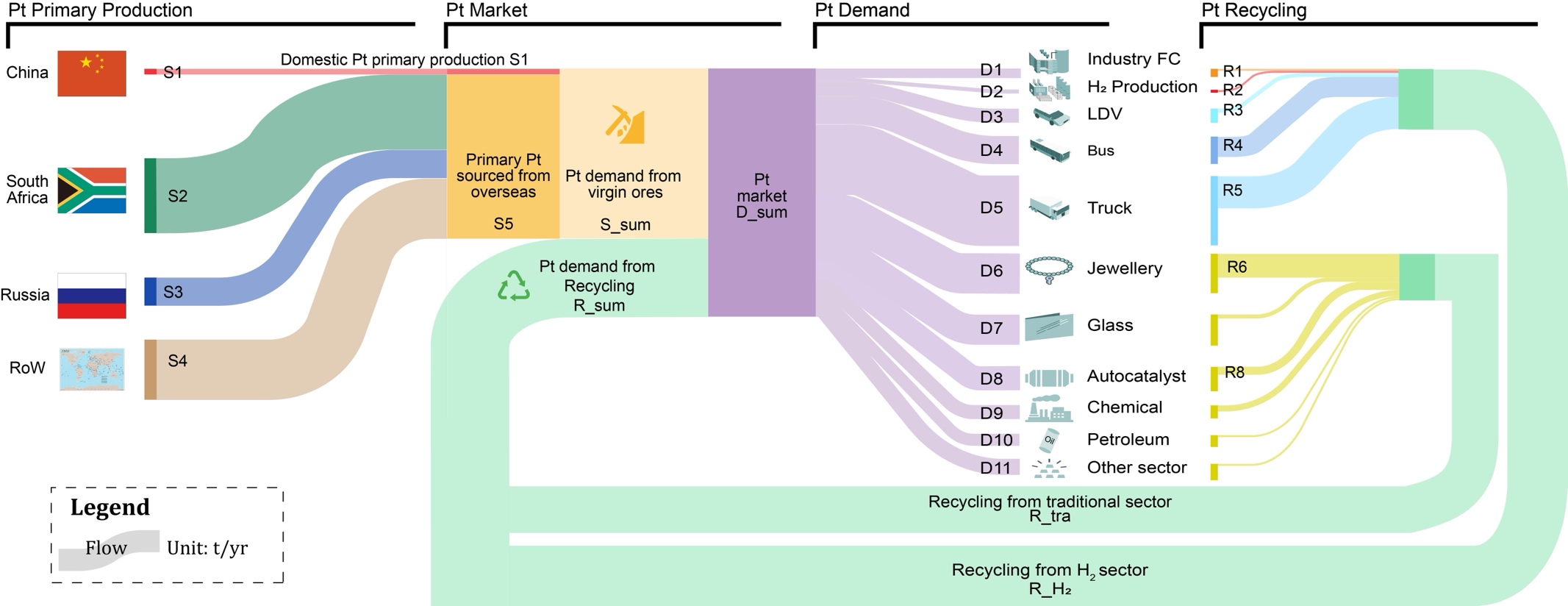


Figure S11 Framework of China's Pt cycle in the future

Table S15 Detailed explanation of the calculation of each flow in China's Pt cycle.

| Flow | Description | Calculation | Data source |
| --- | --- | --- | --- |
| D1 | Pt demand for Industry FC | Stock-driven model | Calculation in this study |
| D2 | Pt demand for H_2_ Production | Stock-driven model | Calculation in this study |
| D3 | Pt demand for LDV | Stock-driven model | Calculation in this study |
| D4 | Pt demand for Bus | Stock-driven model | Calculation in this study |
| D5 | Pt demand for Truck | Stock-driven model | Calculation in this study |
| D6 | Pt demand for Jewellery | Statistics | SFA Oxford[44] |
| D7 | Pt demand for Glass | Statistics | SFA Oxford[44] |
| D8 | Pt demand for Autocatalyst | Stock-driven model | Calculation in this study |
| D9 | Pt demand for Chemical | Statistics | SFA Oxford[44] |
| D10 | Pt demand for Petroleum | Statistics | SFA Oxford[44] |
| D11 | Pt demand for Other sector | Statistics | SFA Oxford[44] |
| D_sum | Total Pt demand | D_sum=D1+…+D11 | Mass balance |
| R1 | Recycling of Industry FC | Stock-driven model | Calculation in this study |
| R2 | Recycling of H_2_ Production | Stock-driven model | Calculation in this study |
| R3 | Recycling of LDV | Stock-driven model | Calculation in this study |
| R4 | Recycling of Bus | Stock-driven model | Calculation in this study |
| R5 | Recycling of Truck | Stock-driven model | Calculation in this study |
| R6 | Pt demand for Jewellery | Statistics | SFA Oxford[44] |
| R8 | Pt demand for Autocatalyst | Stock-driven model | Calculation in this study |
| R_tra | Recycling from traditional sectors | Statistics with the updated R8 | SFA Oxford[44] |
| R_H_2_ | Recycling from H_2_ sector | R_H_2_=R1+R2+R3+R4+R5 | Mass balance |
| R_sum | Pt demand from Recycling | R_sum=R_tra+R_H_2_ | Mass balance |
| S_sum | Pt demand from virgin ores | S_sum=D_sum-R_sum | Mass balance |
| S1 | China's domestic Pt primary production | Statistics | BGS[42] |
| S5 | Primary Pt sourced from overseas | S5=S_sum-S1 | Mass balance |
| S2 | Primary Pt sourced from South Africa | S2=S5*Import share_ZA | Mass balance |
| S3 | Primary Pt sourced from Russia | S3=S5*Import share_RUS | Mass balance |
| S4 | Primary Pt sourced from Rest of World | S4=S5*Import share_RoW | Mass balance |

## *Supplemental Result 1*

***Pt use in Auto catalytic***

Auto catalytic is an increasingly important sector for platinum with the environmental regulations becomes strict. To capture the trend of Pt demand in auto catalytic, Table S16 shows the Pt loading of each auto type of catalytic from (Hao et al., 2019)[10], and Table S17 shows our assumption of the market share of auto catalytic, and the model results are compline with the Pt demand in China in recent years.

Table S16 Pt loadings of Pt in auto catalytic (Unit: g/kW)

| **Vehicle Type** | **Auto catalytic type** | **Pt loading** |
| --- | --- | --- |
| Light duty-gasoline vehicle | TWC | 0 |
|  | GPF | 0.008 |
| Light duty-  diesel vehicle | DOC | 0.011 |
|  | DPF | 0.033 |
|  | SCRF | 0 |
| Heavy duty-diesel vehicle | DOC | 0.024 |
|  | DPF | 0.089 |
|  | SCRF | 0 |

Table S17 Market share of auto catalytic in ICEVs

| **Vehicle Type** | **Vehicle Size Class** | **Market share** |
| --- | --- | --- |
| Light duty-gasoline vehicle | Compact Car | 100% TWC+GPF |
|  | Large Car and SUV |  |
|  | Mini Car |  |
|  | Multipurpose Vehicle |  |
|  | Subcompact Car |  |
| Light duty-diesel vehicle | Light Bus | 50% DOC+DPF 50% DOC+SCRF |
|  | Light Truck |  |
|  | Middle Truck |  |
| Heavy duty-diesel vehicle | Heavy Bus | 100% DOC+SCRF |
|  | Heavy Truck |  |

With the upgrade of China's emission regulations to China VI vehicle emission standards, auto catalysts equipped in new vehicles prefer to be capable of meeting the National VI b emission regulations rather than only met the lower standard National VI a emission limits to avoid the risk of non-compliance with emissions.

China VI vehicle emission standards require significantly stricter limit values on NO_X_, C_x_H_y_ and Particulate Matter (PM). Gasoline vehicles need to add a gasoline particulate filter (GPF) to the existing Three-Way Catalytic (TWC) to increase the capture of suspended particles. Diesel vehicles calls for the introduction of Selective Catalytic Reduction (SCR) and Diesel Particulate Filler (DPF) with the existing Diesel Oxidation Catalyst (DOC). At the same time, a new trend is captured that platinum-containing DPF in diesel vehicles is being replaced by platinum-free selective catalytic reduction traps (SCRF), which can not only reduce NOx emissions, but also filter particulate emissions.

We assume the combination of TWC and GPF cover the whole gasoil vehicles (including compact car, large car& SUV, mini car, MPV and subcompact car) market, and the combination of DOC and SCRF occupy the whole heavy diesel market (including heavy trunk and heavy bus). As well, the combination of DOC and SCRF and the combination of DOC and DPF split the market share of light diesel (including light trunk, light bus and middle trunk) market equally. Based on this auto catalytic technology market share assumption and the Pt loading of each technology, the content of platinum per vehicle are quantified and can be found in Table S18.

Table S18 Pt content per vehicle of different type of ICEVs (Unit: g/vehicle)

| **Vehicle Type** | **Pt content per vehicle** |
| --- | --- |
| Compact Car | 0.8 |
| Large Car and SUV | 0.96 |
| Mini Car | 0.48 |
| Multipurpose Vehicle | 0.96 |
| Subcompact Car | 0.72 |
| Heavy Truck | 7.2 |
| Light Truck | 4.13 |
| Middle Truck | 5.5 |
| Heavy Bus | 4.8 |
| Light Bus | 4.13 |

As environmental regulations tighten, Pt demand for automotive catalysts grows from 5.8t in 2019 to 11.9t in 2021 (JM). The result of our model shows that Pt demand for auto catalysts during 2021 to 2060 will reach 228.1 t, 216.7 t and 217.3 t in CN, 1.5℃ and BAU scenario (Figure S12). Considering the recycling as a supplement source of Pt supply, the net demand for Pt should be 68.9, 63.9 and 65.7 ton in CN, 1.5℃ and BAU scenario, respectively, which means auto catalytic sector, which means taking Pt from the auto catalyst sector to the fuel cell sector is unrealistic. Based our calculation, in nearly 2040, Pt demand for auto catalytic would be negative, which means recycling would meet the Pt demand in this sector. It is worth stating that the number of ICEVs would be higher in the CN scenario because biomass liquids production will have a great increase in this scenario.

Recycling may not be ideal considering over 90% Pt used in auto catalytic are consumed by diesel vehicles. Compared to gasoline auto catalytic, diesel auto catalytic contains lower content of palladium and rhodium, thus the lower value of platinum group metals will diminish the interest of recyclers. At the same time, diesel auto catalytic contains silicon carbide and most smelters need to blend the diesel catalyst with other Pt containing end-of-life products to reduce the carbon content to smeltable levels.


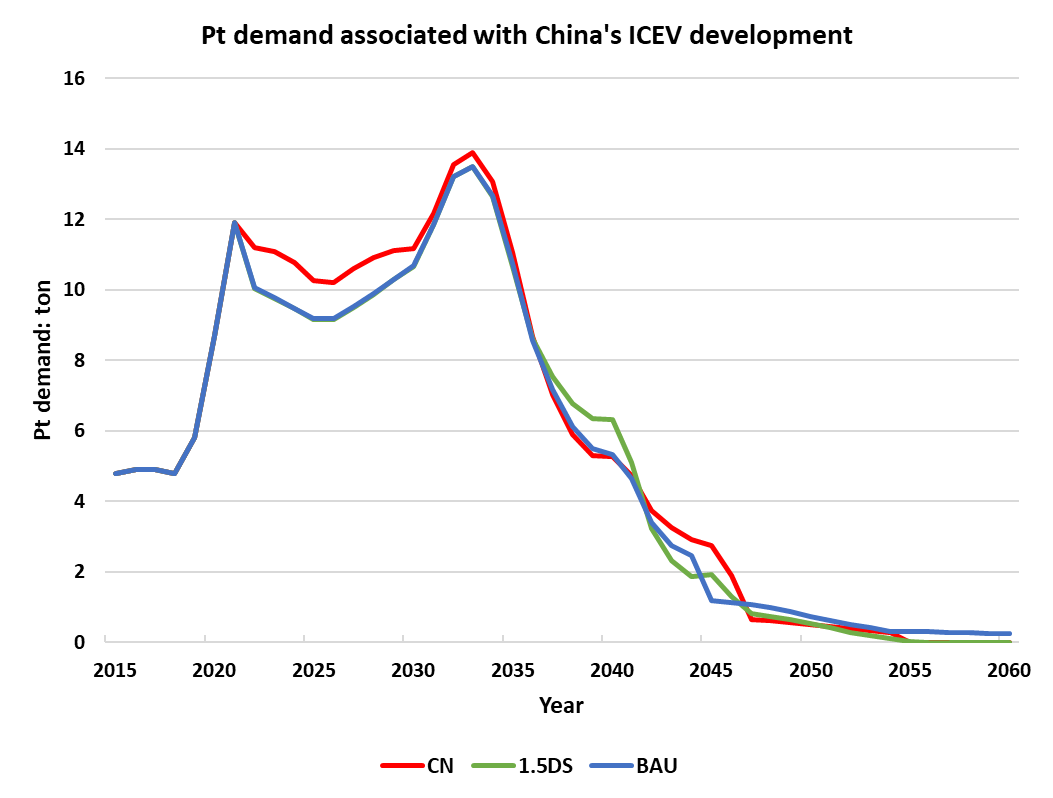


**Figure S12** Pt demand associated with China’s ICEV development.

## *Supplemental Result 2*

***Ir use in PEMWE***

According to literature[50,51], two Ir loading of PEMWE scenarios are collected. As Figure S13 presents, the conservative scenario (a) represents the current mainstream technology level, assuming Ir catalyst loading of 0.33 g/kW from 2015 to 2060. In contrast, the innovative scenario(b) symbolizes the transition from current mainstream technology to advanced laboratory technologies, assuming Ir catalyst loading of 0.33 g/kW from 2020 reduced to 0.05 g/ kW by 2035, and then remains at this level through to 2060.


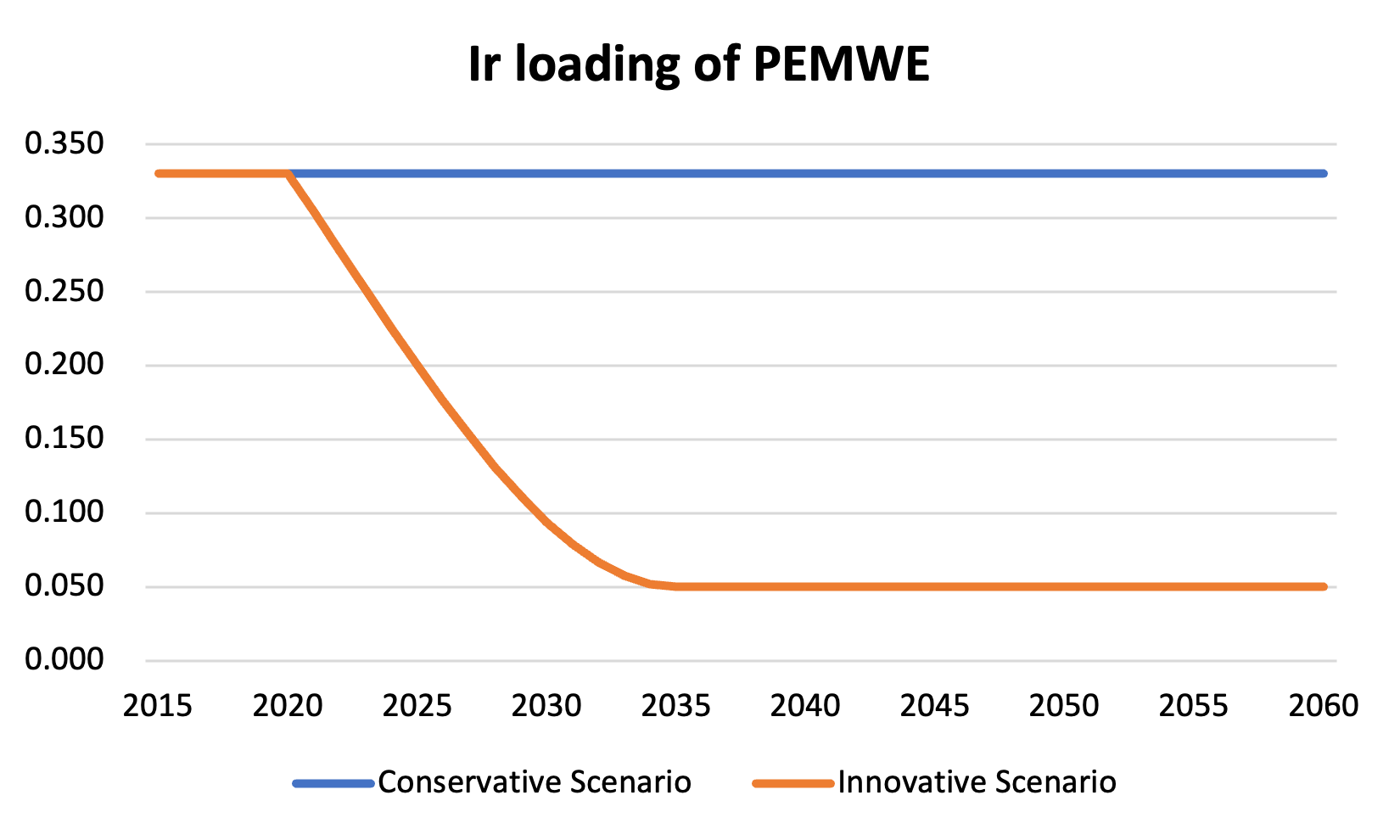


**Figure S13** Ir loading of PEMWE. (a) Conservative scenario; (b) Innovative scenario.

The future Ir demand associated with PEMWE development is displayed in Figure S14.


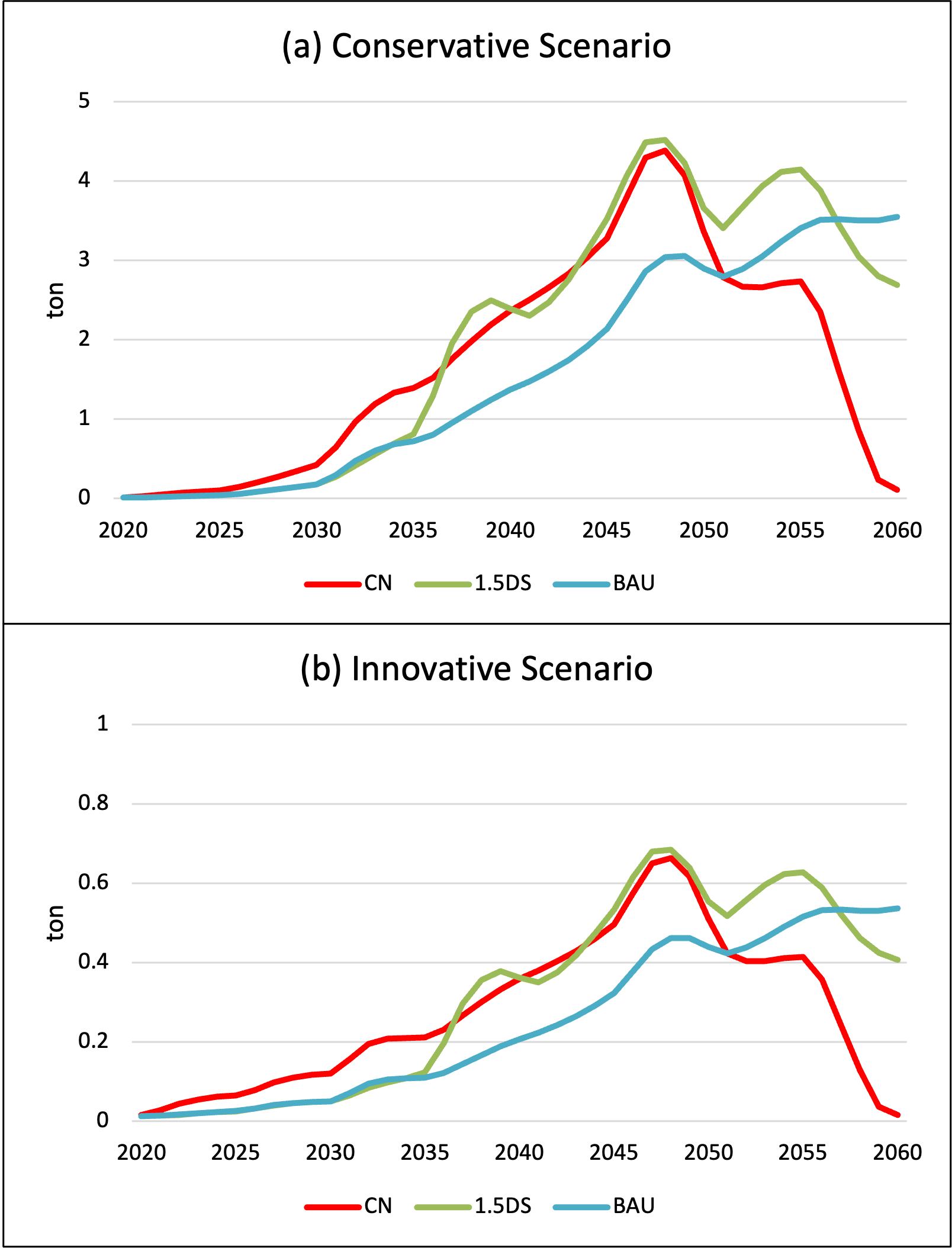


**Figure S14** Ir demand associated with China’s PEMWE market. (a) Conservative scenario; (b) Innovative scenario.

## *Supplemental Sensitivity Analysis*

The material flow framework is the key module to reduce the uncertainties of the bridged MEC Nexus. This study examines the sensitivity of key material flow paraments using one-variable-at-a-time approach (OAT).

In our analysis model, there are a series of factors causing the divergences between those projections including (a) lifetimes of hydrogen infrastructures, (b) all Pt loadings, (c) Pt loadings of PAFC, (d) Pt loadings of PEMFC, (e) Pt loading of PEMWE, (f) Power output of FCEVs, (g) market share of Pt-containing FC in industry energy use (excluding CHP), (h) market share of Pt-containing FC in industry CHP, (i) market share of PEMWE and (g) recycle rate.

The objective of our sensitivity analysis is to confirm that our conclusions are robust to variations in the four exogenous variable sets. We select CN scenario, 1.5°C scenario and BAU scenario as proxies to present the results of our sensitivity analysis and extend these scenario into sixteen “sensitivity analysis” scenarios (i.e. base case, (1) lifetimes of hydrogen infrastructures with a 5% extension, (2) lifetimes of hydrogen infrastructures with a 5% reduction,(3) all Pt loadings with a 5% increase, (4) all Pt loadings with a 5% reduction, (5) Pt loadings of PAFC with a 5% increase, (6) Pt loadings of PAFC with a 5% reduction, (7) Pt loadings of PEMFC with a 5% increase, (8) Pt loadings of PEMFC with a 5% reduction, (9) Pt loading of PEMWE with a 5% increase, (10) Pt loading of PEMWE with a 5% reduction, (11) power output of FCEVs with a 5% increase, (12) power output of FCEVs with a 5% reduction, (13) market share of Pt-containing FC in industry energy use (excluding CHP) with a 5% increase, (14) market share of Pt-containing FC in industry energy use (excluding CHP) with a 5% reduction, (15) market share of Pt-containing FC in industry CHP with a 5% increase, (16) market share of Pt-containing FC in industry CHP with a 5% reduction, (17) market share of PEMWE with a 5% reduction, (18) recycle rate with a 5% increase and (19) recycle rate with a 5% reduction.

The sensitivity analysis results of China’s total Pt demand, total amount of recycled Pt and the total amount of primary Pt during 2021-2060 are shown in Figure S15, Figure S15 and Figure S15. The result shows that factors related to the FCEVs Pt intensity, e.g. lifetimes of hydrogen infrastructures, all Pt loadings change, Pt loadings of PEMFC and power output of FCEVs, have relatively greater impacts. Adjustments in recycling rates may not alter total demand but can impact the demand for platinum from virgin ores by influencing the amount of platinum obtained through recycling.


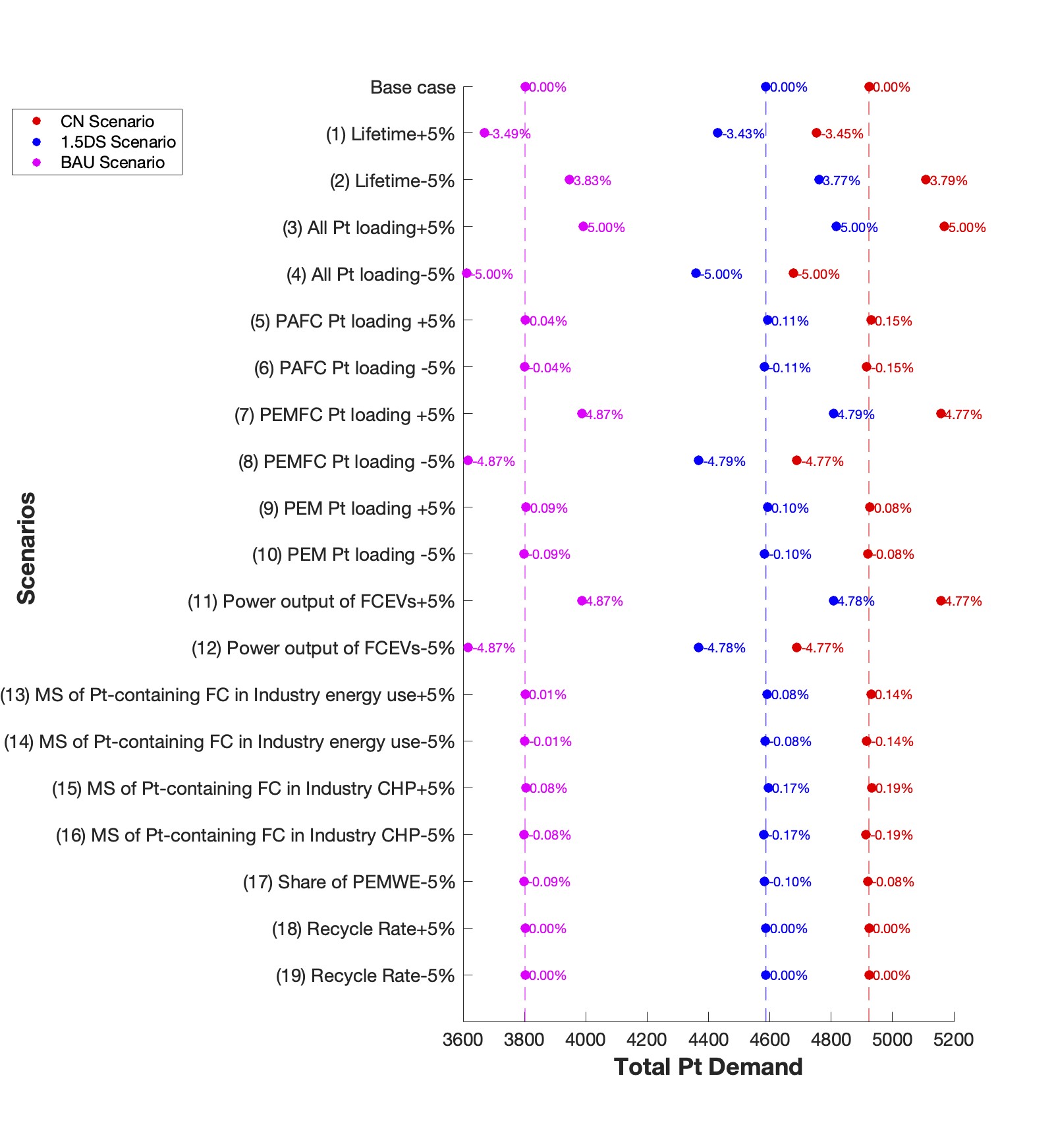


Figure S15 Sensitivity analysis result of key variables on China' total Pt demand from 2021 to 2060. Three reference lines indicate the total Pt demand during 2021 to 2060 in the CN scenario, 1.5°C scenario and BAU scenario. The labelled numbers indicate the percentage of deviation from the baseline scenario for each “sensitivity analysis” scenarios.


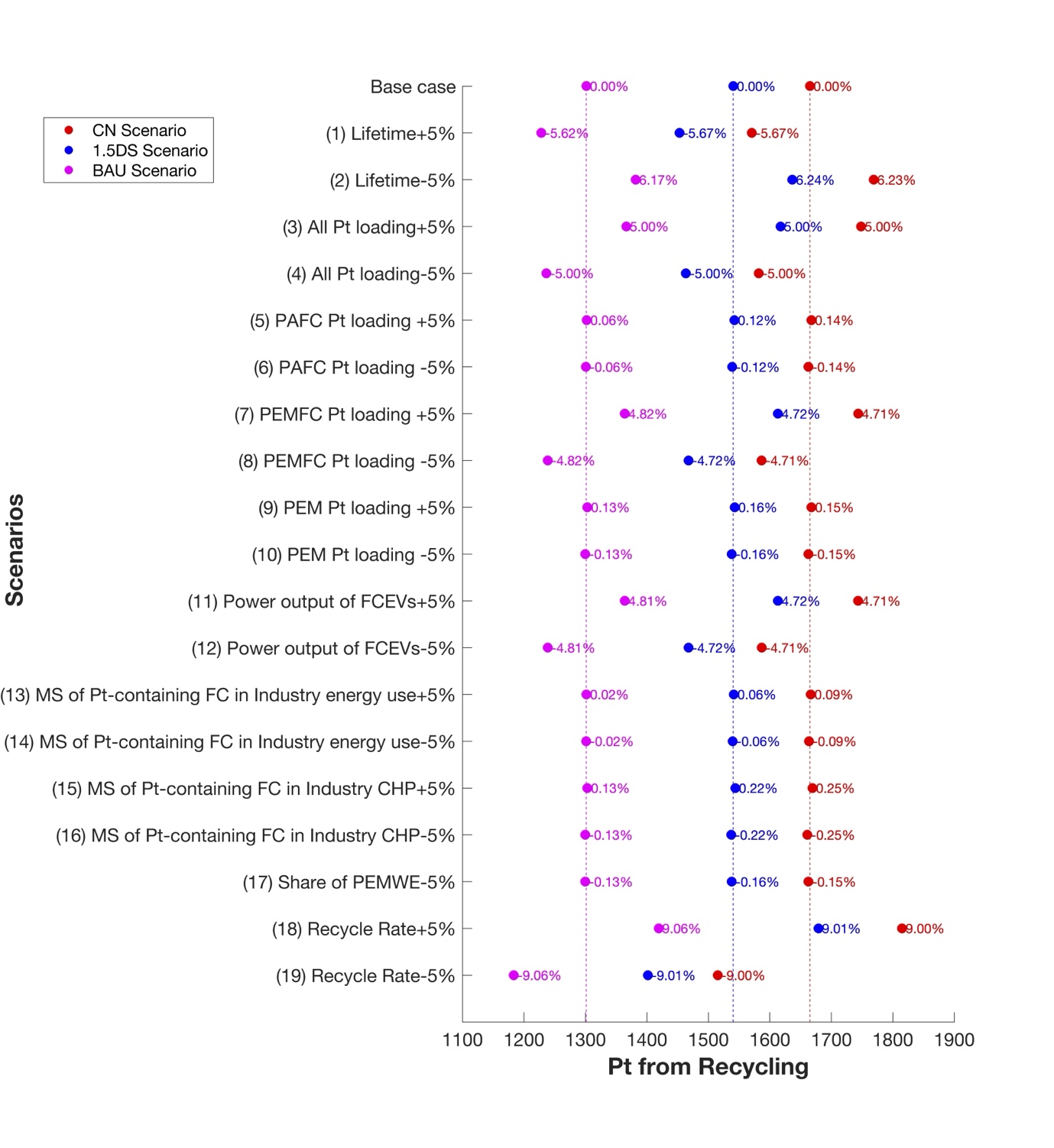
 **Figure S16 Sensitivity analysis result of key variables on total amount of recycling Pt from 2021 to 2060.** Three reference lines indicate the total Pt demand from virgin ores during 2021 to 2060 in the CN scenario, 1.5°C scenario and BAU scenario. The labelled numbers indicate the percentage of deviation from the baseline scenario for each “sensitivity analysis” scenarios.


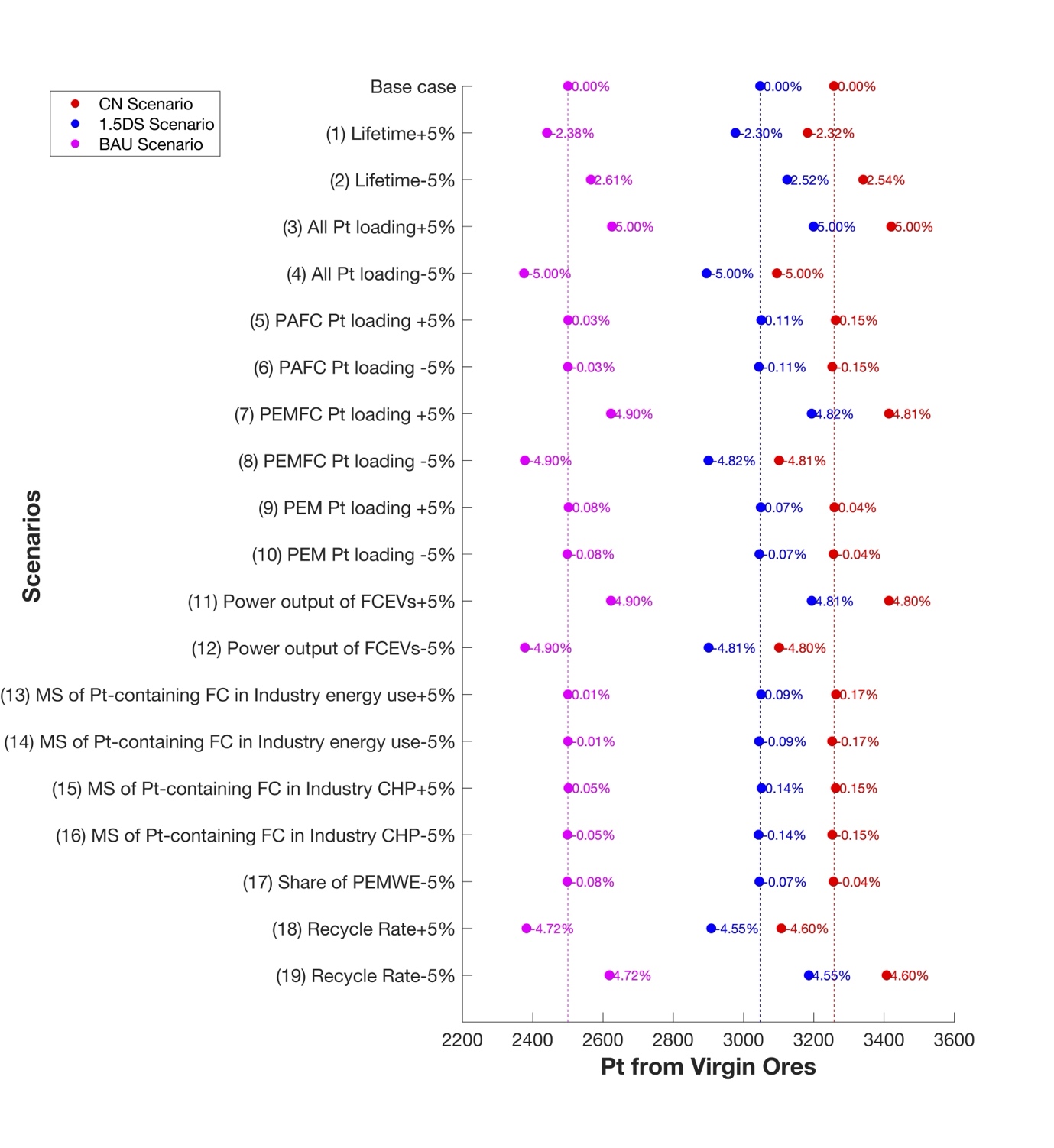


**Figure S17 Sensitivity analysis result of key variables on total amount of primary Pt from 2021 to 2060.** Three reference lines indicate the total Pt demand from virgin ores during 2021 to 2060 in the CN scenario, 1.5°C scenario and BAU scenario. The labelled numbers indicate the percentage of deviation from the baseline scenario for each “sensitivity analysis” scenarios.

## *Abbreviated letter index table*

Table S19 List of abbreviations.

| Abbreviation | Full Name |
| --- | --- |
| H_2_ | Hydrogen |
| Pt | Platinum |
| LDV | Light-duty Vehicles |
| CN | Carbon Neutrality |
| BAU | Business-As-Usual |
| CCS | Carbon Capture and Storage |
| FC | Fuel Cell |
| FCEV | Fuel Cell Electric Vehicle |
| BEV | Battery Electric Vehicle |
| PEM | Polymer Electrolyte Membrane |
| PAFC | Phosphoric Acid Fuel Cell |
| SOFC | Solid Oxide Fuel Cell |
| HRS | Hydrogen Refuelling Station |
| MCFC | Molten Carbonate Fuel Cell |
| PEMFC | Proton Exchange Membrane Fuel Cell |
| CHP | Combined Heat and Power |
| CR | collection rate |
| ZA | South Africa |
| RUS | Russia |
| RoW | Rest of World |
| EOL-RR | end-of-life recycling rate |
| ICEV | Internal combustion engine vehicle |
| MPV | Multi-Purpose Vehicle |

#

# Supplemental References

1. E4Tech. *The Fuel Cell Industry Review 2020*.

2. Bareiß, K., de la Rua, C., Möckl, M. and Hamacher, T. Life cycle assessment of hydrogen from proton exchange membrane water electrolysis in future energy systems. *Appl Energy*, 2019, **237**, 862–872. https://doi.org/https://doi.org/10.1016/j.apenergy.2019.01.001

3. Carmo, M., Fritz, D. L., Mergel, J. and Stolten, D. A comprehensive review on PEM water electrolysis. *Int J Hydrogen Energy*, 2013, **38**(12), 4901–4934.

4. Smolinka, T., Wiebke, N., Sterchele, P., Lehner, F. and Jansen, M. *Sudie_IndWEDe-Industrialisierung der Wasserelektrolyse in Deutschland: Chancen und Herausforderungen für nachhaltigen Wasserstoff für Verkehr, Strom und Wärme*. Nationale Organisation Wasserstoff- und Brennstoffzellentechnologie–NOW GmbH, Berlin, 2018.

5. Bernt, M., Siebel, A. and Gasteiger, H. A. Analysis of voltage losses in PEM water electrolyzers with low platinum group metal loadings. *J Electrochem Soc*, 2018, **165**(5), F305.

6. Rasmussen, K. D., Wenzel, H., Bangs, C., Petavratzi, E. and Liu, G. Platinum Demand and Potential Bottlenecks in the Global Green Transition: A Dynamic Material Flow Analysis. *Environ Sci Technol*, 2019, No. September.

7. Mayyas, A. T., Ruth, M. F., Pivovar, B. S., Bender, G. and Wipke, K. B. *Manufacturing cost analysis for proton exchange membrane water electrolyzers*.

8. Ministry of Industry and Information Technology. *Technology Roadmap for Energy-Saving and New Energy Vehicles*. China Machine Press, Beijing, 2016.

9. New Energy and Industrial Technology Development Organization. *Fuel cell and hydrogen technology roadmap*. China Machine Press, Beijing, 2017.

10. Hao, H., Geng, Y., Tate, J. E., Liu, F., Sun, X., Mu, Z., et al. Securing Platinum-Group Metals for Transport Low-Carbon Transition. *One Earth*, 2019, **1**(1), 117–125. https://doi.org/https://doi.org/10.1016/j.oneear.2019.08.012

11. Remick, R. and Wheeler, D. *Molten Carbonate and Phosphoric Acid Stationary Fuel Cells: Overview and Gap Analysis*. NREL, United States, 2010.

12. Staffell, I. Zero carbon infinite COP heat from fuel cell CHP. *Appl Energy*, 2015, **147**, 373–385.

13. Sun, Y., Delucchi, M. and Ogden, J. The impact of widespread deployment of fuel cell vehicles on platinum demand and price. *Int J Hydrogen Energy*, 2011, **36**(17), 11116–11127. https://doi.org/10.1016/j.ijhydene.2011.05.157

14. SFA Oxford. *The PGM Quarterly Report*.

15. Metals Focus. Platinum & Palladium Focus https://www.metalsfocus.com/product/platinum-palladium-focus/ (accessed 2022-03-01).

16. World Platinum Investment Council. *Platinum Quarterly (PQ)*.

17. Macrotrend. Platinum Prices-Interactive Historical Chart https://www.macrotrends.net/2540/platinum-prices-historical-chart-data.

18. Hotelling, H. The economics of exhaustible resources. *Journal of political Economy*, 1931, **39**(2), 137–175.

19. Weitzman, M. L. On the" environmental" discount rate. *J Environ Econ Manage*, 1994, **26**(2), 200–209.

20. Stern, N. *Stern Review: The economics of climate change*. Cambridge University Press, 2007.

21. Du Preez, M. The discount rate for public sector conservation projects in South Africa. *African development review*, 2004, **16**(3), 456–471.

22. Hydrogen Council. *Hydrogen for Net Zero-A Critical Cost-competitive Energy Vector*.

23. IEA. *An Energy Sector Roadmap to Carbon Neutrality in China*.

24. China Hydrogen Alliance. *White Paper on China Hydrogen and Fuel Cell Industry 2020*.

25. Shell. *Shell Scenarios: Sky - Meeting the goals of the Paris agreement*.

26. BP. *BP Energy Outlook 2022*.

27. Deloitte. *Green hydrogen: Energizing the path to net zero*. (accessed 2023-10-25)

28. Raymond, T., Sterck, E. and Clifford, B. *PLATINUM ESSENTIALS: Fuel cell electric vehicles are forecast to drive material long-term demand growth for platinum*. WPIC, London, 2022.

29. Tong, X., Dai, H., Lu, P., Zhang, A. and Ma, T. Saving global platinum demand while achieving carbon neutrality in the passenger transport sector: linking material flow analysis with integrated assessment model. *Resour Conserv Recycl*, 2022, **179**, 106110. https://doi.org/https://doi.org/10.1016/j.resconrec.2021.106110

30. Alonso, E., Field, F. R. and Kirchain, R. E. Platinum Availability for Future Automotive Technologies. *Environ Sci Technol*, 2012, **46**(23), 12986–12993. https://doi.org/10.1021/es301110e

31. Rasmussen, K. D., Wenzel, H., Bangs, C., Petavratzi, E. and Liu, G. Platinum Demand and Potential Bottlenecks in the Global Green Transition: A Dynamic Material Flow Analysis. *Environ Sci Technol*, 2019, **53**(19), 11541–11551. https://doi.org/10.1021/acs.est.9b01912

32. Elshkaki, A. An analysis of future platinum resources, emissions and waste streams using a system dynamic model of its intentional and non-intentional flows and stocks. *Resources Policy*, 2013, **38**(3), 241–251. https://doi.org/10.1016/j.resourpol.2013.04.002

33. Fuhrman, J., Clarens, A. F., McJeon, H., Patel, P., Ou, Y., Doney, S. C., et al. The role of negative emissions in meeting China’s 2060 carbon neutrality goal. *Oxford Open Climate Change*, 2021, **1**(1), kgab004. https://doi.org/10.1093/oxfclm/kgab004

34. Abdelkareem, M. A., Elsaid, K., Wilberforce, T., Kamil, M., Sayed, E. T. and Olabi, A. Environmental aspects of fuel cells: A review. *Science of the Total Environment*, 2021, **752**. https://doi.org/10.1016/j.scitotenv.2020.141803

35. Calvin, K., Patel, P., Clarke, L., Asrar, G., Bond-Lamberty, B., Cui, R. Y., et al. GCAM v5. 1: representing the linkages between energy, water, land, climate, and economic systems. *Geosci Model Dev*, 2019, **12**(2), 677–698.

36. Zhang, Y., Zhang, Z., Miao, Y., Liu, X. and Wu, Y. *Report on the development of China’s hydrogen industry (in Chinese)*.

37. Nassar, N. T. *Global stocks and flows, losses, and recoveries of platinum-group elements*.

38. Graedel, T. E., Allwood, J., Birat, J., Buchert, M., Hagelüken, C., Reck, B. K., et al. What do we know about metal recycling rates? *J Ind Ecol*, 2011, **15**(3), 355–366.

39. Graedel, T. E., Allwood, J., Birat, J.-P., Buchert, M., Hagelüken, C., Reck, B. K., et al. *Recycling rates of metals: a status report*. United Nations Environment Programme, 2011.

40. Duclos, L., Chattot, R., Dubau, L., Thivel, P. X., Mandil, G., Laforest, V., et al. Closing the loop: Life cycle assessment and optimization of a PEMFC platinum-based catalyst recycling process. *Green Chemistry*, 2020, **22**(6), 1919–1933. https://doi.org/10.1039/c9gc03630j

41. Staffell, I., Scamman, D., Abad, A. V., Balcombe, P., Dodds, P. E., Ekins, P., et al. The role of hydrogen and fuel cells in the global energy system. *Energy Environ Sci*, 2019, **12**(2), 463–491.

42. Idoine, N. E., Raycraft, E. R., Shaw, R. A., Hobbs, S. F., Deady, E. A., Everett, P., et al. *World Mineral Production 2016-20*. British Geological Survey, Keyworth, Nottingham, 2022.

43. U.S. Geological Survey. *Mineral commodity summaries 2022*.

44. SFA Oxford. *Platinum Standard*.

45. Ministry of Natural Resources, P. *China Mineral Resources*. GEOLOGICAL PUBLISHING HOUSE, Beijing, 2021.

46. LBMA. LBMA Precious Metal Prices: Platinum https://www.lbma.org.uk/cn/prices-and-data#/%0A (accessed 2021-05-01).

47. UN Comtrade. United Nations commodity trade statistics database https://comtrade.un.org/ (accessed 2022-03-01).

48. Nansai, K., Nakajima, K., Kagawa, S., Kondo, Y., Suh, S., Shigetomi, Y., et al. Global Flows of Critical Metals Necessary for Low-Carbon Technologies: The Case of Neodymium, Cobalt, and Platinum. *Environ Sci Technol*, 2014, **48**, 1391−1400.

49. Bernt, M., Siebel, A. and Gasteiger, H. A. Analysis of voltage losses in PEM water electrolyzers with low platinum group metal loadings. *J Electrochem Soc*, 2018, **165**(5), F305.

50. Hydrogen and Fuel Cell Technologies Office. *DOE Technical Targets for Hydrogen Production from Electrolysis*. (accessed 2022-09-25)

51. Minke, C., Suermann, M., Bensmann, B. and Hanke-Rauschenbach, R. Is iridium demand a potential bottleneck in the realization of large-scale PEM water electrolysis? *Int J Hydrogen Energy*, 2021, **46**(46), 23581–23590. https://doi.org/https://doi.org/10.1016/j.ijhydene.2021.04.174
